# A new non-aggregative splicing isoform of human Tau is decreased in Alzheimer’s disease

**DOI:** 10.1007/s00401-021-02317-z

**Published:** 2021-05-02

**Authors:** Vega García-Escudero, Daniel Ruiz-Gabarre, Ricardo Gargini, Mar Pérez, Esther García, Raquel Cuadros, Ivó H. Hernández, Jorge R. Cabrera, Ramón García-Escudero, José J. Lucas, Félix Hernández, Jesús Ávila

**Affiliations:** 1 Departamento de Anatomía, Histología y Neurociencia, School of Medicine, Autonoma de Madrid University (UAM), Arzobispo Morcillo, 4, 28029 Madrid, Spain; 2 Graduate Program in Neuroscience, Autonoma de Madrid University (UAM), Arzobispo Morcillo, 4, 28029 Madrid, Spain; 3 Centro de Biología Molecular “Severo Ochoa” (CSIC-UAM). Nicolás Cabrera, 1. Cantoblanco, 28049 Madrid, Spain; 4 Neurooncology Unit, Instituto de Salud Carlos III-UFIEC, 28220 Madrid, Spain; 5 Unidad de Investigación, Fundación Hospital de Jove, 33290 Gijón, Spain; 6 Molecular Oncology Unit, CIEMAT, Ave Complutense, 40, 28040 Madrid, Spain; 7 Hospital 12 Octubre Research Institute/CIEMAT, Madrid, Spain; 8 Centro de Investigación Biomédica en Red de Cancer (CIBERONC), Valderrebollo, 5, 28031 Madrid, Spain; 9 Networking Research Center on Neurodegenerative Diseases (CIBERNED), Instituto de Salud Carlos III, 28031 Madrid, Spain

**Keywords:** Alzheimer’s disease, Tau, Tauopathies, Truncation, Alternative splicing, Intron retention

## Abstract

Tauopathies, including Alzheimer’s disease (AD) and frontotemporal lobar degeneration with Tau pathology (FTLD-tau), are a group of neurodegenerative disorders characterized by Tau hyperphosphorylation. Post-translational modifications of Tau such as phosphorylation and truncation have been demonstrated to be an essential step in the molecular pathogenesis of these tauopathies. In this work, we demonstrate the existence of a new, human-specific truncated form of Tau generated by intron 12 retention in human neuroblastoma cells and, to a higher extent, in human RNA brain samples, using qPCR and further confirming the results on a larger database of human RNA-seq samples. Diminished protein levels of this new Tau isoform are found by Westernblotting in Alzheimer’s patients’ brains (Braak I *n* = 3; Braak II *n* = 6, Braak III *n* = 3, Braak IV *n* = 1, and Braak V *n* = 10, Braak VI *n* = 8) with respect to non-demented control subjects (*n* = 9), suggesting that the lack of this truncated isoform may play an important role in the pathology. This new Tau isoform exhibits similar post-transcriptional modifications by phosphorylation and affinity for microtubule binding, but more interestingly, is less prone to aggregate than other Tau isoforms. Finally, we present evidence suggesting this new Tau isoform could be linked to the inhibition of GSK3β, which would mediate intron 12 retention by modulating the serine/arginine rich splicing factor 2 (SRSF2). Our results show the existence of an important new isoform of Tau and suggest that further research on this less aggregation-prone Tau may help to develop future therapies for Alzheimer’s disease and other tauopathies.

**Supplementary Information:**

The online version contains supplementary material available at 10.1007/s00401-021-02317-z.

## Introduction

Tau is a microtubule-associated protein (MAP) that acts as a neurite microtubule stabilizer [9, 61]. Tau overexpression, especially in its modified forms (i.e. phosphorylated, aggregated or truncated) may result in a toxic gain of function [5]. Human Tau protein isoforms are expressed from a unique gene (*MAPT*) located at chromosome 17 [30, 42] and has at least 16 exons [2, 3]. Exons 1, 4, 5, 7, 9, 11, 12 and 13 are constitutive while the others are subjected to alternative splicing [2, 60]. Importantly, over 90% of human genes allegedly undergo alternative splicing [59], although recent evidence point out that this may not be the main mechanism to explain proteome complexity [55]. However, there is robust evidence for certain genes being subjected to alternative splicing, yielding well-studied spliced variants, being *MAPT* one of the most relevant [1, 55]. Noteworthy, aberrant splicing has been associated with numerous diseases, including Alzheimer’s disease and other age-related disorders [51]. In accordance with that, mutations altering alternative splicing of exon 10 of *MAPT* cause some tauopathies, including FTDP-17 [16, 25, 32], and may be associated with others such as Huntington’s disease, whose patients display a four-repeat tauopathy with nuclear rods [19].

Truncated Tau proteins lead to a toxic gain of function, promoting abnormal microtubule assembly and inducing aggregation, being a key feature of Alzheimer's disease, especially in the sporadic form [43, 65].

Tau truncation can take place at N-terminal or C-terminal regions [6, 22, 41, 44, 46, 48, 52, 63, 64]. Recently, a truncated toxic Tau fragment raised upon asparagine endopeptidase (AEP) cleavage, has been reported in mice model of AD and elderly and Alzheimer’s disease human brains [63]. This toxic fragment contains residues 1–368 of the human Tau molecule and the last residue (asparagine 368) coincides with the end of exon 12.

Here, we present evidence of a new, human-specific truncated form of Tau similar to Tau 1–368 generated by intron 12 retention in human neuroblastoma cell lines and brain. An analogous mechanism of truncation has been described for a toxic huntingtin fragment [52]. This intron-retaining RNA species would generate a truncated Tau protein similar to the one resulting from AEP cleavage, but followed by 18 extra amino acids, which dramatically reduces its ability to aggregate.

Strikingly, the resulting Tau truncated isoform is reduced in Alzheimer’s disease patients’ brain, especially in later stages of the disease, in contrast to increasing total Tau levels. Despite the similarity of its sequence to that of the AEP truncated isoform, this new Tau isoform is able to bind to microtubules and is less prone to aggregate, suggesting a beneficial role in the pathology.

## Materials and methods

### Human brain samples

Hippocampal and frontal lateral cortex brain samples from sporadic Alzheimer’s disease patients and control subjects were kindly provided by Dr. A. Rabano from Banco de Tejidos (Fundación CIEN, Instituto de Salud Carlos III, Madrid, Spain). Based on quantitative pathological features, the Alzheimer’s brain specimens were classified according to Braak stages I (*n* = 3), II (*n* = 6), III (*n* = 4), IV (*n* = 1), V (*n* = 10) and VI (*n* = 8), and non-demented control subjects (*n* = 10) (see Supplementary table 1, online resource for further subject information). Written informed consent premortem was obtained from all patients.

### Nomenclature

Classical nomenclature [2] for *MAPT* gene exon/intron numbers has been used consistently throughout the paper, although some of the databases (such as ENSEMBL: https://www.ensembl.org/index.html) employ a different nomenclature assigning different numbers to exons and introns (according to ENSEMBL, intron retention would take place in intron 13).

As for the new Tau isoform described in this work, the transcript including the translation of intron 12 up to the first stop codon has been named *TIR-MAPT* (*Truncated by Intron Retention* MAPT), while the protein generated from such transcript has been termed W-Tau, due to the appearance of two characteristic tryptophan residues (W) in this isoform, an amino acid that does not appear elsewhere within the Tau molecule.

In addition, Tau truncated by asparagine endopeptidase, as previously described by Zhang et al. [63] is referred to as ET-Tau (*Endopeptidase Truncated Tau*) throughout the text.

### Cell culture

HEK293T (CRL-11268, ATCC), SK-N-MC (HTB-10, ATCC), and SH-SY5Y (CRL-2266, ATCC) cells were cultured in DMEM or MEM supplemented with 10% fetal bovine serum, 2 mM glutamine, non-essential amino acids, 10 U/ml penicillin and 10 µg/ml streptomycin, at 37 °C and 5% CO_2_.

### *TIR-MAPT* and *ET-MAPT* cloning

*TIR-MAPT* transcript was obtained from an SH-SH5Y RNA extract, using specific oligos (TauNt and TauD, see Supplementary table 2, online resource) and was cloned into pBlueScript-SK + (212,205, Agilent Technologies), thanks to a TA-cloning strategy [37]. Using specific oligos that include the appropriate restriction sites (A1 and TIR-T-BglII and ET-T-BglII, see Supplementary table 2, online resource) *TIR-MAPT* and *ET-MAPT* were subcloned into a eukaryotic expression vector pSG5 (216201, Agilent technologies). Similarly, Tau isoforms were cloned into a prokaryotic expression vector pRK172 using specific oligos (TAU-PRK172 fw, TIR-T-pRKpWPI rv and ET-T-pRKpWPI rv; see Supplementary table 2, online resource). After cloning, all vectors were sequenced using the described oligos (Supplementary table 2, online resource).

### RNA-seq data analysis

RNA-seq raw data from 363 samples of 3 brain regions (frontal cortex, dorsolateral prefrontal cortex and hippocampus) of 180 human brain healthy donors were retrieved from the Genotype-Tissue Expression (GTEx) project [36] (Supplementary table 3, online resource). SRA files were converted to FASTQ files and reads were re-mapped to human genome GRCh38 using STAR version 2.5.2a [18]. Gene expression quantification was performed with RSEM version 1.3.1 [35].

**Fig. 1:**
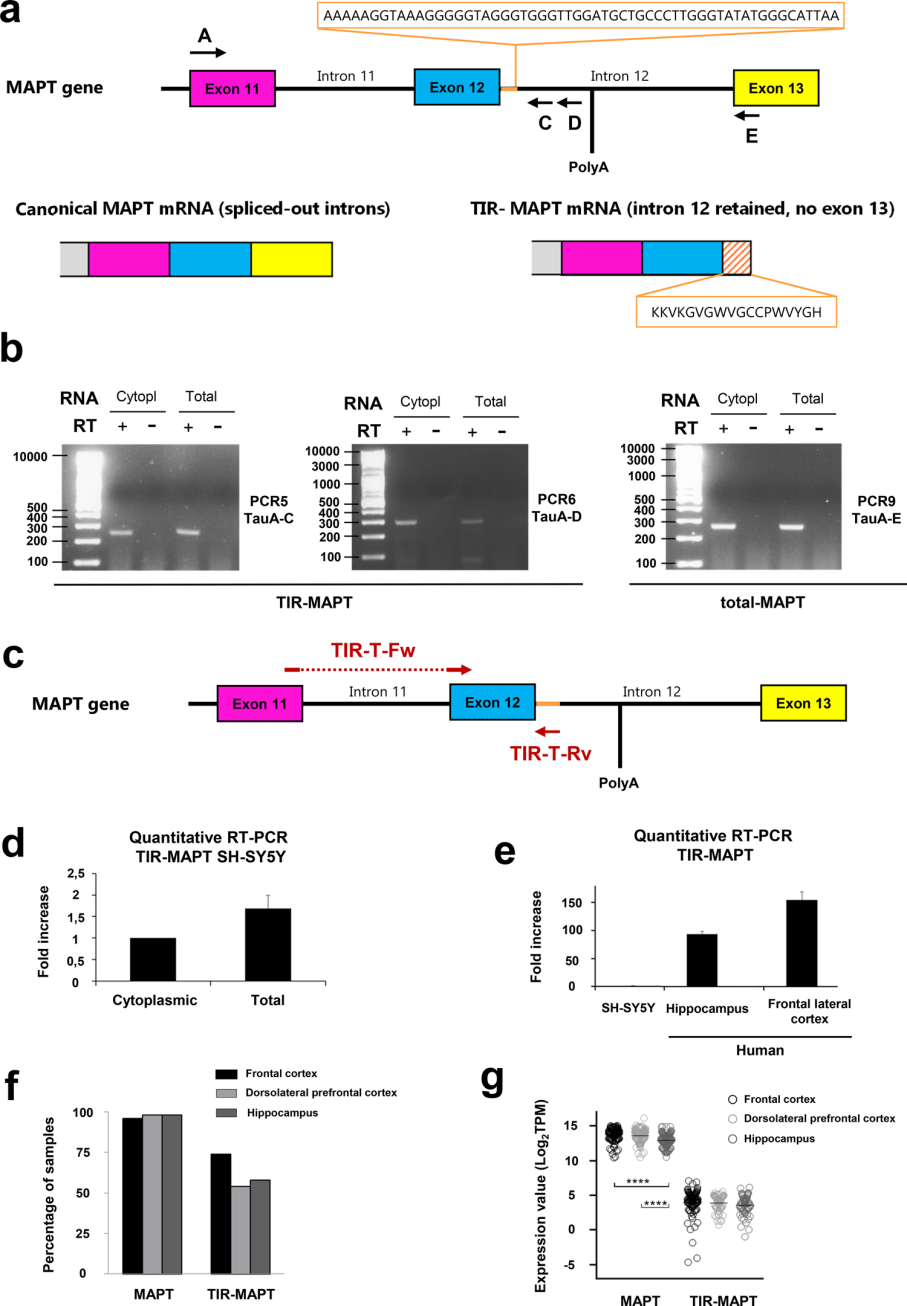
*TIR-MAPT* RNA expression. **a** Schematic representation of the *MAPT* gene and *MAPT* and *TIR-MAPT* mRNAs generated from it. A, C, D and E represent the hybridization sites of the primers designed for semi-quantitative PCR employed for the PCRs in **b** (Supplementary Table 2, online resource). The fragment of intron 12 that would remain upon retention is represented with colored stripes. **b** Representative images of agarose gels showing of semi-quantitative PCR results using total or cytoplasmic-enriched RNA of SH-SY5Y cells. Results showed the existence of RNA species from exon 11 to intron 12 where intron 11 was spliced out (PCR 5 and 6). Controls of the addition (RT+) or no addition of retrotranscriptase (RT−) were included. PCR 9 shows *MAPT* in which both intron 11 and 12 are spliced out. Detailed information of all semi-quantitative PCR combinations and amplicon sizes is provided in Supplementary Table 5, online resource. **c** Schematic representation of the *MAPT* gene including the hybridization sites of the oligos used for quantitative PCR. **d** *TIR*-*MAPT* RNA levels by qPCR in cytoplasmic-enriched fraction or whole extracts (total) of SH-SY5Y cells. **e** Comparison of *TIR-MAPT* level in SH-SY5Y cells and hippocampus and frontal lateral cortex of human brain. Graphs show means and SE of technical triplicates. **f** Percentage of brain samples having expression of *MAPT* or *TIR-MAPT* genes. Data are shown for three different regions within brains (see Supplementary Table 3, online resource). **g** Scatter dot blot of expression values of *MAPT* and *TIR-MAPT* genes in brain regions having TPM (transcripts per kilobase million) > 0. Number of samples **f**, **g**: cortex = 122; frontal cortex = 113; hippocampus = 98. Graphs represent mean and SEM. *p* values were calculated using a *T* test, ****p value ≤ 0.0001

The annotation file was retrieved from GENCODE (gencode.v23.annotation.gtf) and was modified to include a new *MAPT*-related gene (*TIR-MAPT*) whose genome coordinates are chr17:45894382–46018851, which includes part of the intron 12 (chr17:46018731–46018851) as the 3′ end of the gene in the region mapping the oligonucleotide sequence TauD (Supplementary Table 2, online resource and Fig. 1). Gene expression levels were obtained from RSEM as transcripts per kilobase million (TPM) values. Expression levels of *MAPT* and *TIR-MAPT* genes were analyzed per brain region. For those donors with more than one sample in the same brain region, analysis was done only in the sample with highest *MAPT* expression.

### Bacteria culture and Tau purification

Upon cloning, pRK172 vectors encoding different Tau isoforms were transformed in BL21 *E. coli* competent cells by electroporation, from which Tau was purified, as described elsewhere [49]. These bacteria were cultured in LB medium with 100 ng/ml of ampicillin at 37 °C overnight. Bacterial suspensions were transferred to 1L of LB with 100 ng/ml of ampicillin and further incubated at 37 °C, up until optical density readings at 600 nm ranged between 0.6 and 0.8. At this point, 0.4 mM of IPTG was added to each sample so as to trigger transcription, and incubated once again at 37 °C for 2 h. The samples were centrifuged at 2950×*g* at 4 °C for 20 min and pellets were resuspended in Tau buffer, which consists of buffer A (0.1 M MES pH 6.4, 0.5 mM MgCl_2_ and 2 mM EGT) supplemented with 1 mM PMSF; 0.5 NaCl and 5 mM β-mercaptoethanol. Resuspended pellets were sonicated on ice at 24 microns peak to peak (mpp) five times for 1 min, waiting 10 s between repetitions. Upon sonication, samples were centrifuged at 13,850×*g* at 4° C for 10 min and the resultant supernatants were subjected to 5 min at 100° C and 5 min on ice before centrifuging one more time at 13,850×*g* at 4 °C for 30 min. Supernatants were kept and Tris 1 M was added until pH reaches a level of 11, to get rid of residual DNA. Ammonium sulfate 50% was added to the samples and they were incubated agitating at 4 °C for at least 1 h before centrifuging again at 13,850×*g* at 4 °C for 1 h. Pellets containing purified Tau were resuspended in buffer A and the purification process was checked by SDS-PAGE and Coomassie blue staining to check if any other protein bands appeared upon Coomassie blue staining.

### Tau protein quantitation

Protein concentration for each Tau isoform was first determined by means of absorbance measurements corrected by individually calculated extinction coefficients (ε), based on the specific amino acid sequence of each isoform [24] (Supplementary Table 4, online resource). Namely, we measured Tau concentration as indicated by Kundel and collaborators [34]: absorbance at 280 nm (*A*_280_) was measured for each Tau isoform and concentration was determined as the ratio between *A*_280_ and *ε*. Extinction coefficients can be estimated from the amino acid sequence of the protein [24], according to the equation: *ε*_280_ = number of tryptophan residues (W) × 5500 + tyrosine residues × 1490 [17]. Using this equation and the amino acid composition of each Tau isoform, the extinction coefficients were calculated for each one (Supplementary Table 4, online resource). Calculated values for T42 and T30 coincide with those calculated by Kundel [34], which has been typically used for all CNS human Tau isoforms [21], but are not valid for W-Tau isoforms that contain 2 extra tryptophan residues nor for ET-Tau isoforms, which lack 1 tyrosine that appear on the rest of the isoforms. Coomassie blue staining of samples containing the same amount of protein was performed to confirm their concentrations.

### RNA extraction and purification

Either total RNA or cytosolic enriched fraction RNA was purified from cells using RNAeasy Mini Kit (74104, Qiagen) following the protocols described in Qiagen handbook. For brain tissue, previous homogenization using a TissueLyser (Retsch MM300, Qiagen, Hilden, Germany) (30 Hz, 5 min) with 5-mm stainless steel beads (69989, Qiagen) in 700 µl QIAzol Lysis Reagent (79306, Qiagen) was performed. RNA integrity numbers (RIN) were calculated using the Agilent 2100 Bioanalyzer system (Agilent Technologies), and only RNAs with RIN > 5 were used for RT-qPCR.

RNA was purified using RNAeasy Mini Kit (74104, Qiagen) with the following modifications. Cells were pelleted and frozen at -80 °C. Pellets were carefully resuspended in 175 μl of precooled (4 °C) buffer: 50 mM TrisHCl pH 8, 140 mM NaCl, 1.5 mM MgCl_2_, 0.5% (v/v) Nonidet P40 (1.06 g/ml) plus 1 mM DTT just before use and incubated on ice for 5 min. Lysates were centrifuged at 4 °C for 2 min at 300 × g. Supernatants were kept as cytoplasmic-enriched fraction. To each fraction, 600 μl of Buffer RLT were added. After vortexing, 430 μl of ethanol 100% were added to the homogenized lysate. Samples were transferred to RNeasy spin columns, centrifuged for 15 s at 9000×*g* and the flow-through was discarded. Samples were treated with 10 µl of DNase in 70 µl of buffer RDD (RNase-free DNase Set 79254, Qiagen) for 15 min at room temperature. The rest of the extraction was performed following the protocol and RNA was collected in 30 µl of RNAse free water twice.

### Semi-quantitative PCR

Total RNA was purified using RNAeasy Mini Kit (74104, Qiagen) following provider’s guidelines. Cytoplasmic RNA fractions were purified as previously described. Retrotranscription was performed using 40 ng/µl of RNA with the Transcriptor First Strand cDNA Synthesis Kit (04379012001, Roche) using oligo(dT)18 primer. Semi-quantitative PCR was performed using 1 µl of cDNA (0.5 ng/µl) supplemented with 2.5 mM MgCl_2_, 0.2 mM each dNTP, 1.3 M betaine, 0.5 mM of each primer (described in supplementary table 2, online resource) and 0.025 U/ml of GoTaq^®^ Flexi DNA Polymerase (M829, Promega), in the following conditions: 95 °C for 2 min, and 35 cycles (PCR1-8) or 30 cycles (PCR9-13) of 95 °C for 45 s, 58.6 °C for 45 s and 72 °C for 45 s, followed by a final extension of 10 min at 72 °C. PCR combinations are described in supplementary table 5, online resource.

### Detection of Tau levels by quantitative RT-PCR

RNA was retrotranscribed with the Transcriptor First Strand cDNA Synthesis Kit (04379012001, Roche) using 20 ng/µl RNA with oligo(dT)18 primer. Quantitative PCR was performed in a LightCycler480 (Roche) in the following conditions: 50 °C for 2 min, 95 °C for 10 min, and 40 cycles of 95 °C for 15 s and 60 °C for 1 min. Specific intron-spanning oligonucleotides against *MAPT* or *TIR-MAPT* transcript were designed (*MAPT*-E11-E13-fw/rv and TIR-T-fw/rv, respectively; see Supplementary table 2, online resource). Gene expression was normalized to GAPDH expression using TaqMan primer human GAPDH (Hs02758991_g1, Applied Biosystems). For every RT-qPCR experiment, only samples with RIN above 5 were used.

### Detection of Tau levels by Western blot

**Fig. 2:**
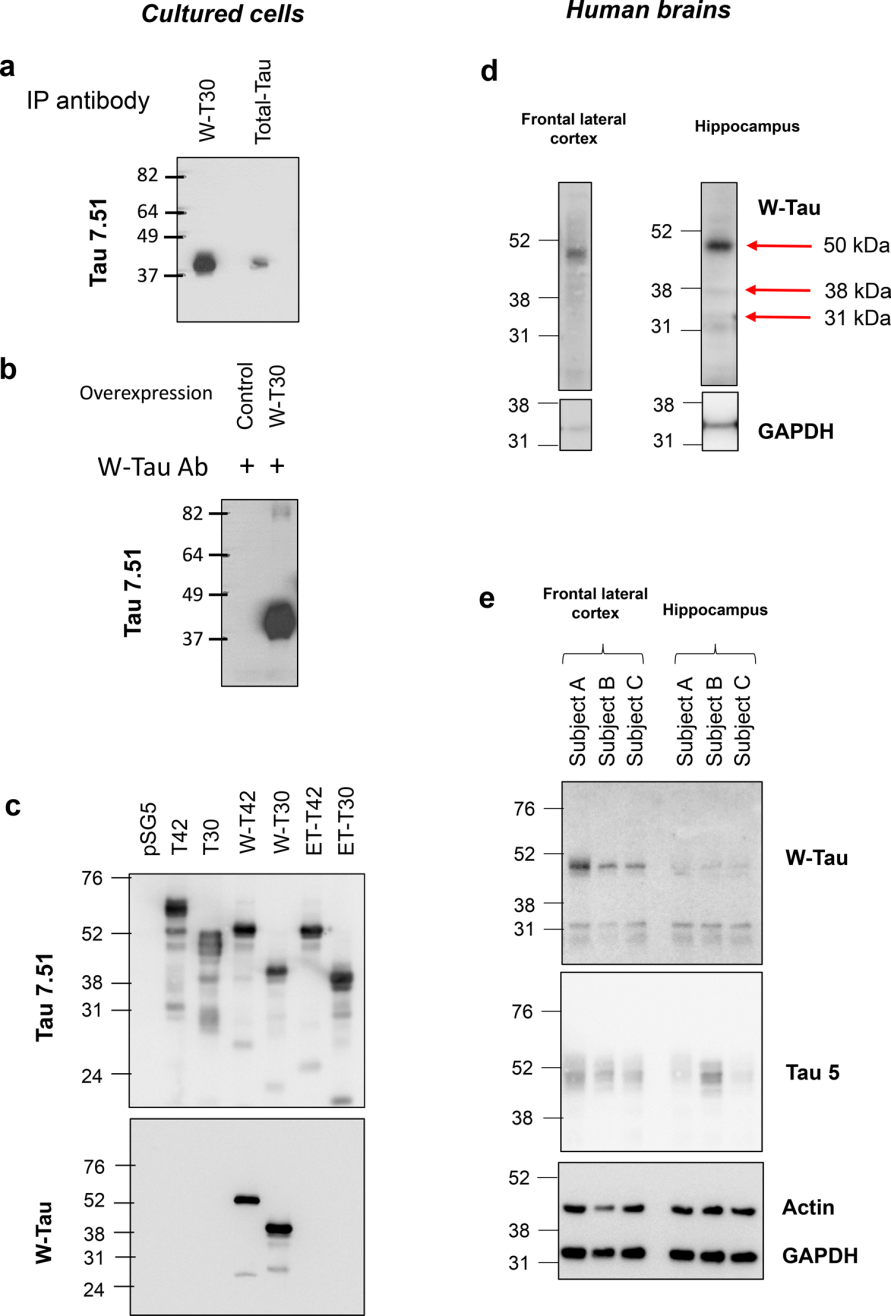
W-Tau antibody validation and protein expression in human brain. **a** Immunoprecipitation assay of HEK239T cells overexpressing W-T30 using W-Tau (Abyntek) or total Tau (NOVUSBIO, NB100-82247) antibodies and Western blot detection of immunoprecipitated Tau using 7.51 antibody. **b** Immunoprecipitation of the same cells overexpressing W-T30 and control untransfected cells using W-Tau antibody and detection of immunoprecipitated Tau using total Tau (Tau 7.51) antibody. **c** HEK293T cells were transfected with eukaryotic expression vectors empty or encoding different *MAPT* isoforms (T42, T30, W-T42, W-T30, ET-42 and ET-T30). Representative Western Blot for Tau 7.51 and W-Tau antibodies proving W-Tau specificity. **d** Western blot detection of W-Tau in frontal lateral cortex and hippocampus of one human subject that show bands at 50 kDa (W-T42), 38 KDa (W-T30), and 31 KDa (W-Tau truncated). **e** Representative Western blot of frontal lateral cortex and hippocampus samples of the same three human subjects (**a**, **b**, **c**) showing the presence of W-Tau and total Tau (Tau5)

**Fig. 3:**
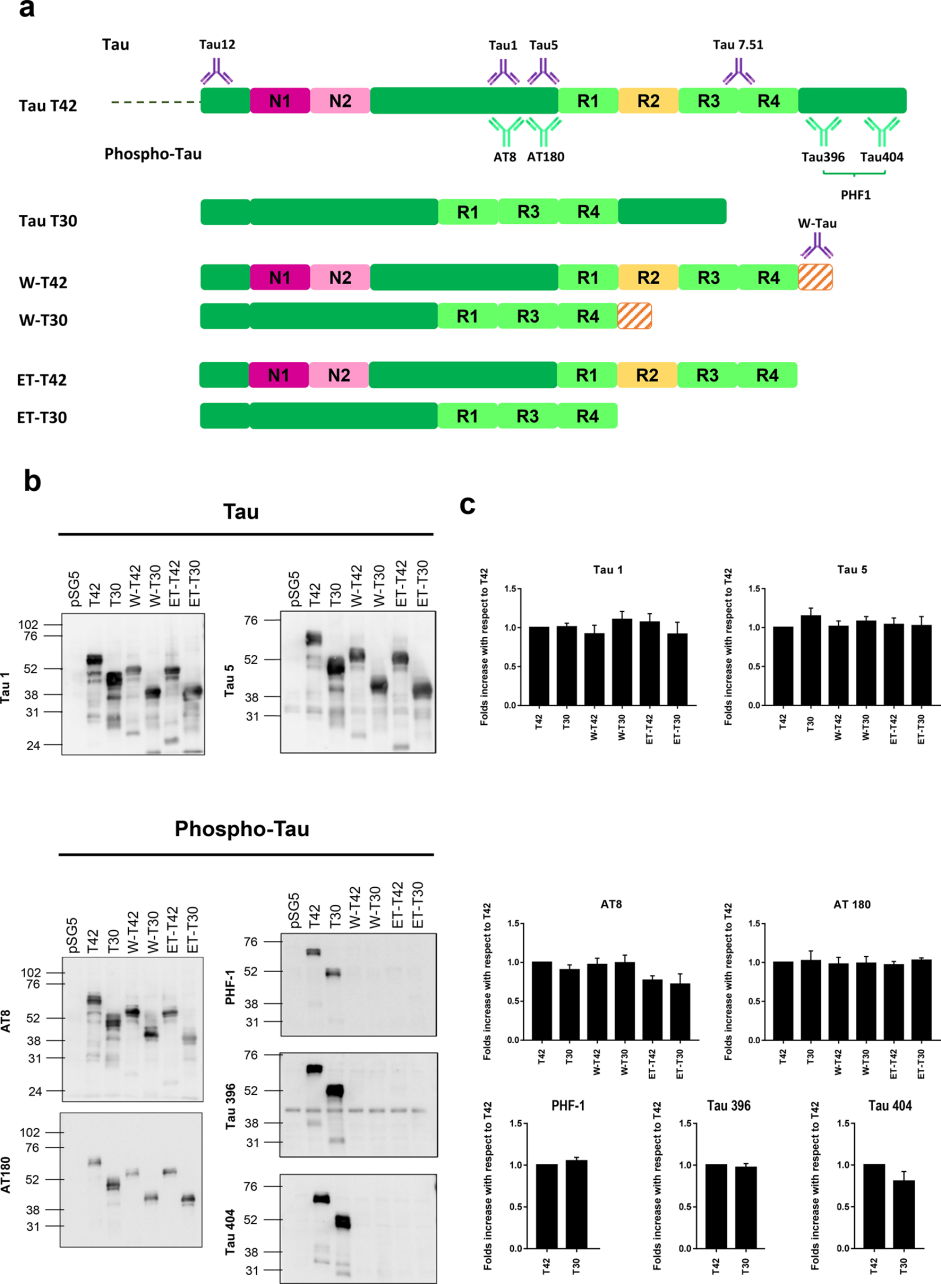
W-Tau phosphorylation pattern. **a** Schematic representation of different Tau protein isoforms: full-length isoform with four repeats (R) and two insertions (N) (T42), full-length isoform with three repeats and no insertions (T30), the truncated by intron retention isoforms W-Tau with four repeats, two insertions and an extra peptide (W-T42) or with three repeats, no insertions and the extra peptide (W-T30); and the correspondent asparagine-endopeptidase-truncated isoforms (ET-T42 and ET-T30). Representation of the antibodies recognizing the Tau molecule at their corresponding epitopes: Antibodies recognize all isoforms of Tau (Tau12 on amino acids 6–18; Tau 5 on amino acids 210–241 and Tau 7.51 on amino acids 315–376) or specific against dephosphorylated Tau in residues Ser195, 198, 199 and 202 (Tau1), and phospho-Tau in residues Ser202/Thr205 (AT8), Thr231 (AT180), Ser404 (Tau404), Ser396 (Tau396) and Ser396/Ser404 (PHF1). W-Tau antibody recognizes the unique peptide present on W-Tau isoforms. **b** Samples from HEK293T cells transfected with the different isoforms were probed with different antibodies for phosphorylated and non-phosphorylated Tau. **c** Quantification of the data of different Tau epitopes with respect to total Tau measured with Tau 7.51 antibody, showing mean and SEM (*n* = 4). One-way ANOVA for multiple comparisons followed by a Kruskal–Wallis test was performed to compare each isoform and T42 full-length level of phosphorylation

**Fig. 4:**
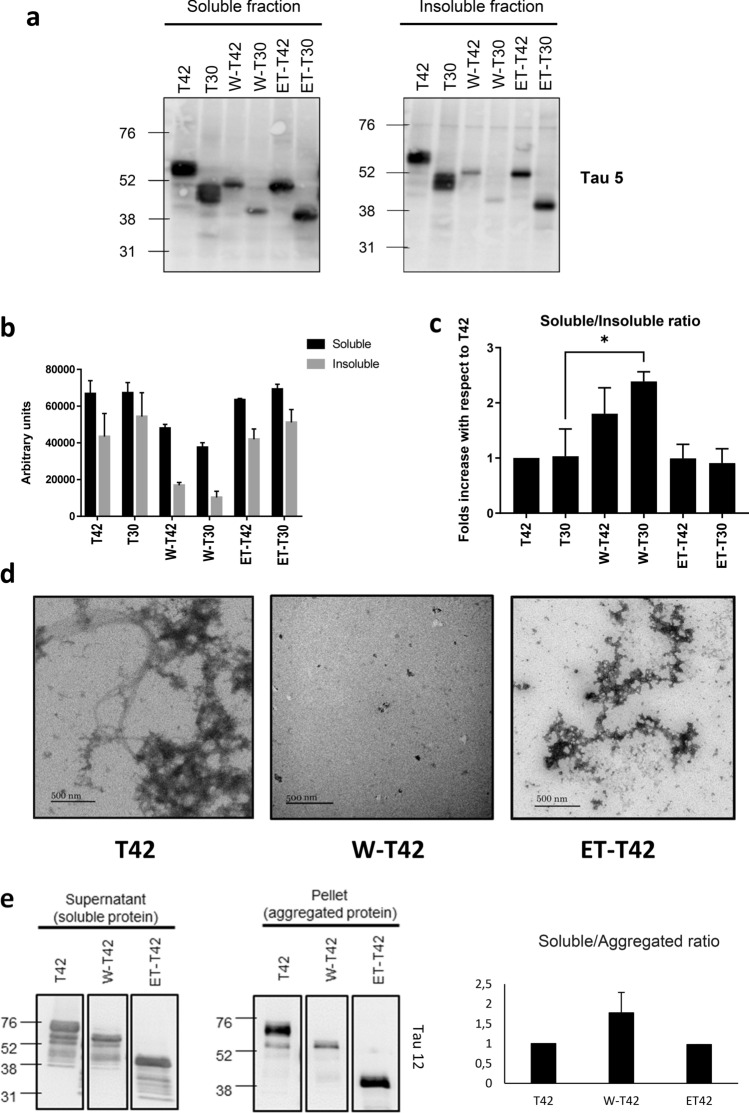
W-Tau aggregation capacity. **a** Representative Western blot of the presence of Tau in 1% sarkosyl-soluble and insoluble cell fractions of HEK293T cell overexpressing different Tau isoforms (T42, T30, W-T42, W-T30, ET-42 and ET-T30) detected with Tau 5 antibody. b, c Quantification of the signal obtained from Tau 5 showing sarkosyl-soluble vs insoluble fractions (**b**) or soluble/insoluble ratio (**c**). Graphs show means and SEM (*N* = 3). One-way ANOVA for multiple comparisons followed by a Kruskal–Wallis test was performed to compare each isoform with the correspondent full-length isoform, *p ≤ 0.05. **d** Representative electron microscopy images of Tau aggregates obtained upon in vitro incubation from partially purified T42, W-T42, and ET-42 extracts from bacteria in the presence of heparin to prompt aggregates formation. Scale bars show 500 nm wide. **e** Western blot showing soluble (supernatant) and aggregated (pellet) protein from the incubation in **d**, upon centrifugation to separate both fractions. Quantification shows the ratio between soluble and aggregated protein

**Fig. 5:**
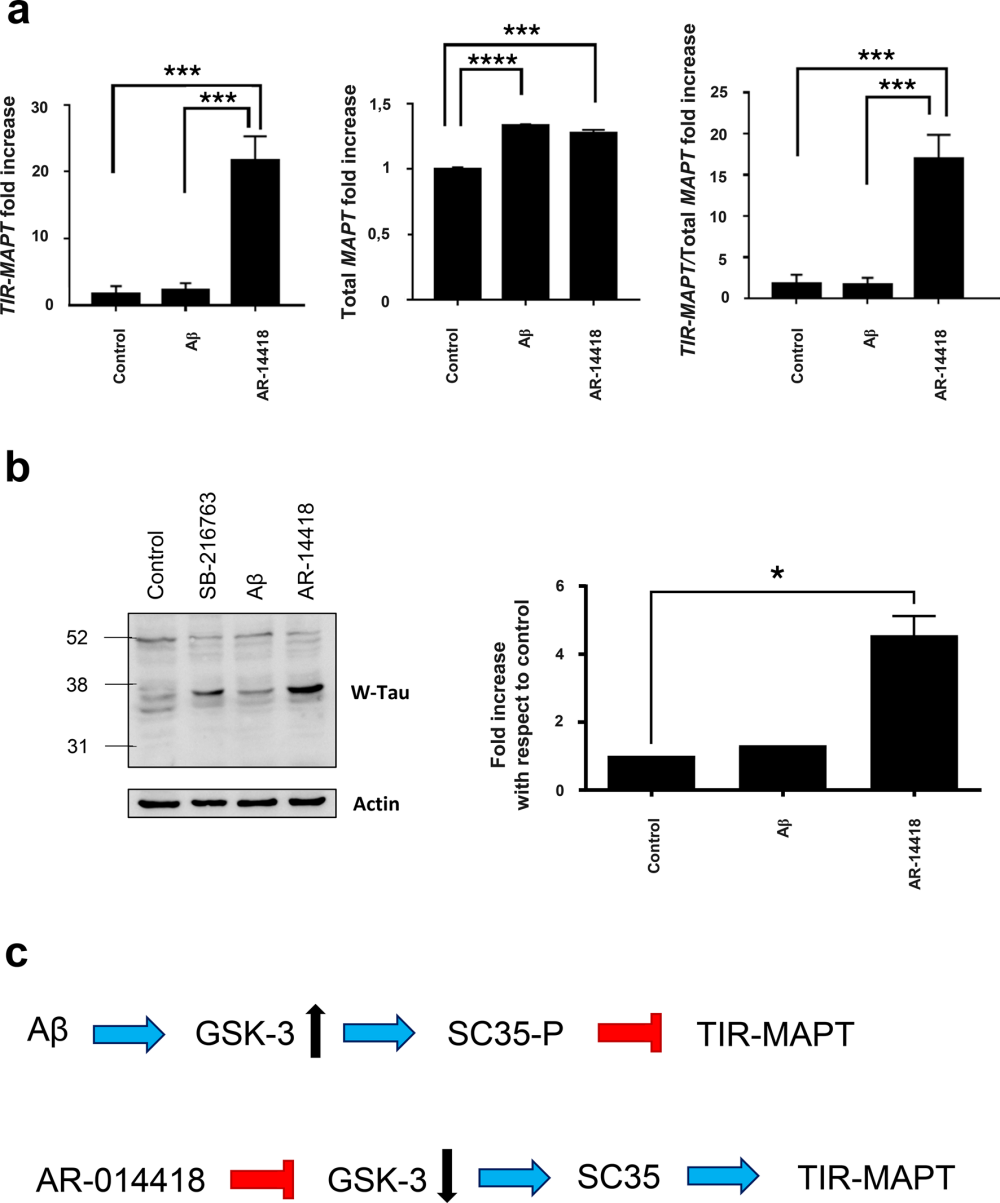
Modulation of *TIR-MAPT* and W-Tau by GSK3. **a**. Detection of mature cytoplasmic RNA levels of *TIR-MAPT*, total *MAPT* and the ratio *TIR-MAPT*/total *MAPT* in SK-N-MC human neuroblastoma cell line in the absence or presence of 5 µg/ml amyloid-β_1–42_ and the GSK3 inhibitor AR-014418 (10 µM) for 24 h. Graphs show mean and SEM. One-way ANOVA and Dunnett’s multiple comparisons test were performed and statistical significance between untreated and cells treated with either Aβ_1–42_ and AR-014418 was given. ***p ≤ 0.001; ****p ≤ 0.0001. **b** Western blot analysis of the protein levels of W-Tau (W-Tau antibody) in SK-N-MC cells treated with SB216763 (25 µM), Aβ_1–42_ (1.1 µM) or AR-14418 (10 µM). Right panel shows the quantification of the signal of complete lanes obtained with W-Tau antibody. Graphs show mean and SEM. One-way ANOVA for multiple comparisons followed by a Kruskal–Wallis test was performed to compare treated and untreated cells. *p ≤ 0.05. **c** Schematic representation of the modulation of *TIR-MAPT* by splicing factor SC35, regulated by GSK3-mediated phosphorylation. Blue arrows represent activation; red, truncated arrows represent inhibition

Frozen brain tissue was homogenized using a TissueLyser (Retsch MM300, Qiagen, Hilden, Germany) (30 Hz, 5 min) with 5-mm stainless steel beads (69,989, Qiagen) in total extraction buffer: 50 mM Tris–HCl pH 7.5, 300 mM NaCl, 0.5% SDS (sodium dodecyl sulphate) and 1% Triton X-100 (Fig. 2d, e and Supplementary Fig. 6b, online resource). The same buffer was also used for cells (Figs. 2b, c, 3b, 4a, 5b and Supplementary Fig. 4b, online resource and Supplementary Fig. 5a, b, online resource). For human cortex brain extracts, a strong lysis buffer (50 mM Tris–HCl pH 7.6, 400 mM NaCl, 1 mM EDTA, 1 mM EGTA, 1% SDS) was also used in Fig. 7b. The homogenates are incubated for 15 min at 95 °C, centrifuged (16,100×*g*, 10 min) and the supernatant was considered the brain extract. Protein concentration was measured using the DC protein assay kit (500-0111, Bio-Rad). From each sample, equal amount of total protein was resolved on a 10% Bis–Tris gel and transferred to nitrocellulose membranes. Blots are probed with the corresponding primary antibodies (see below), followed by horseradish peroxidase-conjugated anti-mouse or anti-rabbit antibody (Dako, Glostrup, Denmark). Protein expression was quantified by measuring the ECL signal with ImageJ software (Fig. 7 and Supplementary Fig. 6, online resource) (http://rsbweb.nih.gov/ij/) or with Quantity One^®^ 1-D analysis software from Bio-Rad.

### Antibodies

Different antibodies were used for the different essays, as indicated in each one. Altogether, Tau 12 (Millipore, MAB2241; amino acids 6-18, diluted 1/1000), Tau 5 (Calbiochem, D00139295, amino acids 210-241, diluted 1/1000), Tau antibody (NOVUSBIO, NB100-82247, epitope around amino acid 231, diluted 1/500) and Tau 7.51 ([44], amino acids 315-376, diluted 1/100) were used as antibodies recognizing all Tau isoforms; Tau 1 (Chemicon, MAB3420, diluted 1/1000) recognizing dephosphorylated residues Ser195, 198, 199 and 202 and AT8 (phospho-Ser202/Thr205; Innogenetics, MN1020, diluted 1/100), AT180 (phospho-Thr231; Innogenetics, MN1040, diluted 1/100), Tau 404 (phospho-Ser404; Life Technologies, 44758G, diluted 1/1000), Tau 396 (phospho-Ser396; Life Technologies, 44752G, diluted 1/1000) and PHF1 (phospho-Ser396/Ser404, kind gift of Peter Davies [26], diluted 1/100) as antibodies recognizing phosphorylated Tau isoforms. In addition, β-actin (SIGMA A5441, diluted 1/20000) or GAPDH (Cell signalling, 2118, diluted 1/1000 antibodies were used as loading control.

A peptide composed of the W-Tau unique sequence (KKVKGVGWVGCCPWVYGH) (W-Tau peptide) was synthesised and a polyclonal IgG antibody against that peptide was obtained from Abyntek (Bizkaia, Spain), using New Zealand rabbit as host strain. This antibody was named W-Tau antibody and its specificity was tested (diluted 1/1000) to ensure it reacts exclusively with TIR-Tau but not with other Tau isoforms (Fig. 2a–c).

Immunoprecipitation was performed with W-Tau and total Tau (NOVUSBIO, NB100-82247) antibodies using SantaCruz Immunoprecipitation Kit (SC-2003) using 500 µg of whole cell extracts and following provider’s guidelines.

### Tau phosphorylation determination

HEK293T cells were transfected with pSG5 plasmids (2 µg/p60) encoding different Tau isoforms using Lipofectamine and Plus reagents following instructions of the supplier (#18324 and #11514, respectively, Life Technologies). 48 h after transfection, cells were collected, pelleted and homogenized in 200 µl of total extraction buffer. Protein concentrations were measured using the DC protein assay kit (500-0111, Bio-Rad) and samples containing equivalent amounts of protein were analyzed by Western blot, as previously described, using nitrocellulose membranes. Immunodetection was performed with antibodies Tau 7.51 and Tau 5 for total Tau, Tau 1 for dephosphorylated Tau and AT8, AT180, Tau 404, Tau 396 and PHF1 for phosphorylated Tau.

### Tau solubility determination

Once again, HEK293T cells were transfected with pSG5 plasmids (2 µg/p60) encoding different Tau isoforms using Lipofectamine and Plus reagents (Life Technologies). After 48 h, cells were collected, pelleted and homogenized in 150 µl of lysis buffer (50 mM Tris–HCl (pH 7,4), 150 mM NaCl, 20 mM NaF, 1 mM Na_3_VO_4_, 0.5 mM MgSO_4_; supplemented with protease inhibitors cocktail, 04693159001 Roche) at 4 °C. Homogenates were centrifuged at 27,000×*g* for 20 min at 4 °C. Pellets were discarded and 1% sarkosyl was added to the supernatants, being incubated for 1.5 h agitating at room temperature. Upon incubation, samples were centrifuged at 150,000×*g* during 45 min at 4 °C. Supernatants were kept as sarkosyl-soluble fraction and pellets were resuspended in 100 µl of a mix of total extraction buffer and loading buffer (1:1) and considered sarkosyl-insoluble fraction.

Results were further confirmed by carrying out a similar protocol to measure Triton X-100 solubility. Namely, pelleted cells were homogenized in 500 µl of lysis buffer (1% Triton X-100, 50 mM Tris–HCl pH 7, 100 mM NaCl and 1 mM EDTA) and incubated 20 min at 4 °C. The samples were centrifuged at 18,000×*g* for 5 min and the supernatants were kept as soluble fraction. Pellets were resuspended in total extraction buffer (see point 11 of methods) and considered Triton-insoluble fraction.

In both cases, equivalent volumes of each sample were then analyzed by Westernblotting.

### Tau in vitro aggregation determination

Tau aggregates were grown for the different Tau isoforms by vapor diffusion in hanging drops in the standard way used for protein crystallizations [15]. The different Tau isoforms were purified from bacteria as described above and their concentration was estimated by measuring absorbance at 280 nm and taking into account individually calculated extinction coefficients for each isoform (Supplementary table 4, online resource) and confirmed by means of Coomassie Blue staining. Equivalent quantities of each isoform were added to the corresponding volume of buffer A (0.1 M MES pH 6.4, 0.5 mM MgCl_2_ and 2 mM EGT) plus 50 mM NaCl in the presence of heparin, reaching a concentration of 1 mg/ml. The reservoir contained 0.2 M NaCl in buffer A. Aggregates were obtained after incubation for 10 days at room temperature (see also [49]). An aliquot was kept for electron microscopy analysis, and the rest of the samples were centrifuged in Airfuga at 28 lb per square inch (psi) for 30 min at room temperature to separate soluble and aggregated protein. The fractionated proteins were characterized by electrophoresis and Western blot.

### Tau microtubule-binding capacity assay

For microtubule preparation, the brains of 12 2-month-old C57BL/6J mice were homogenized with a potter in isotonic buffer (0.32 M sucrose, 1 mM EGTA, 1 mM MgCl_2_, 10 mM phosphate buffer pH 7 and 1 mM PMSF) at 4 °C. Supernatant was collected after 40 min ultracentrifugation at 100,000×*g* at 4 °C in an Optima L-100 XP Ultracentrifuge (Beckman Coulter). 100 μl of this supernatant were incubated with equal quantity of protein for the different Tau isoforms, purified from bacteria as mentioned above during 30 min at 37 °C, in the presence of 30% glycerol, 1 mM PMSF and 1 mM GTP to promote tubulin polymerization. After that, samples were ultracentrifuged for 60 min at 100,000×*g* at 25 °C.

Equivalent volumes of pellets containing microtubules and microtubule-bound Tau were analyzed by Western Blot, using Tau 5 and tubulin antibodies and the ratio between Tau 5 and tubulin signal was calculated for each isoform as their microtubule-binding ratio, normalized to the corresponding full-length isoform.

### GSK3 modulation

SK-N-MC human neuroblastoma cells (ATCC^®^ HTB-10™) were treated with GSK3 inhibitors SB216763 (25 µM, GlaxoSmithKline compounds [13]) and AR-14418 (10 µM, AstraZeneca compound [7]) or Aβ_1–42_ (1.1 µM; Neosystem Laboratoire, Strasbourg, France) for 24 h and whole cell extracts were used for Western blot analysis, probing the blots with W-Tau antibody. From those cells treated with AR-14418 and Aβ_1-42_, RNA-enriched cytoplasmic fraction was also retrieved and *TIR-MAPT* RNA levels were assessed by quantitative RT-PCR as previously described.

### Splicing factor binding site prediction analysis

Exonic and intronic human *MAPT* sequences were retrieved from ENSEMBL (ENSG00000186868). Exon 12–exon 13 and exon 12–intron 12 sequences (Exon 13–exon 14 and exon 13–intron 13, according to ENSEMBL nomenclature of Tau exons/introns) were analyzed using ESEFinder 3.0 (http://exon.cshl.edu/ESE/) [53]. ESEFinder settings were adjusted to show binding sites with score 4.0 or higher. The analysis was completed by a detailed bibliographic search comparing SRSF2 binding sites described in Masaki et al., 2019 and Cavaloc et al., 1999 [11, 38] with junctions *MAPT* Exon 12–exon 13 and exon 12–intron 12 sequences.

### Statistical analysis

Quantitative data, represented as mean ± SD or SEM, were compared between groups using the two-tailed Student’s *t* test. For multiple comparisons, one-way ANOVA and Dunnett's test were performed to compare each isoform and the correspondent full-length isoform. When the distribution of the data was not Gaussian a nonparametric Kruskal–Wallis test was used. The differences are given with their corresponding statistical significance or *p* value, which is the probability that the difference occurred merely by chance under the null hypothesis (*p ≤ 0.05; **p ≤ 0.01; ***p ≤ 0.001; ****p ≤ 0.0001; N.S. not significant). The method used for each experiment is specified in the corresponding figure legend.

## Results

### New alternative splicing variant of *MAPT* present in mature human transcriptome

Recently, the presence of new protein isoforms generated by intron retention in neurodegenerative diseases such as Alzheimer’s disease [45] and Huntington’s disease [52] has been described. This, together with evidence of an asparagine-endopeptidase-truncated Tau isoform [63], whose cleavage point coincides with the end of exon 12 led us to seek possible intron retention events within the intron between exons 12 and 13 (intron 12). Curiously, human intron 12 includes a canonical stop codon sequence followed by a polyadenylation canonical site, so intron 12-containing species would be translated as a truncated protein similar to the truncated isoform mediated by AEP (ET-Tau), with 18 more amino acids, which we have named W-Tau (Fig. 1a). Importantly, this isoform would be human-specific, since this polyadenylation site cannot be found in other species, such as mice.

We could detect intron 12 retaining RNA species in which intron 11 was spliced out in SH-SY5Y mature (polyA positive) total RNA and cytoplasmic-enriched fraction (Fig. 1b, Supplementary Fig. 1 and Supplementary Tables 2 and 5, online resources). This was observed by means of semi-quantitative PCR using an exon 11-matching forward oligo and intron 12 reverse oligos (Fig. 1a, b, Supplementary Table 5, online resource, PCR 5 and 6). As expected, we could also detect the equivalent amplicon using an exon 12-matching reverse oligo in which intron 12 was spliced out (Fig. 1a, b, Supplementary Table 5, online resource, PCR 9). Additionally, we could observe similar results using an exon 12 forward oligo and intron 12 reverse oligos (Supplementary Fig. 1a, b, Supplementary Table 5 PCR 1 and 2, online resources). Genomic DNA contamination was ruled out using intron-spanning forward oligos (Supplementary Fig. 1a, b and Supplementary Table 5, PCR 3, 4, 7 and 8; online resources). Curiously, intron-spanning primers matching exon 10 to detect RNA species containing intron 12 never detected these as mature cytoplasmic polyA positive RNA (Supplementary Fig. 1a, b and Supplementary Table 5, PCR 7 and 8; online resources). Exon 10 is spliced out in Tau isoforms with three repeats (Tau 3R) and it has been previously described that proliferative SH-SY5Y cells mainly express these Tau isoforms [57], so detection of RNA species with partial inclusion of exon 10 was not expected a priori. As a control, mature polyA positive RNA species including only exons were found in our samples (Supplementary Fig. 1a, b and Supplementary Table 5, PCR 10–12; online resource**s**), even detecting transcripts that include exon 10 (Tau 4R), although with decreased efficiency (Supplementary Fig. 1a, b and Supplementary table 5, PCR 12; online resources). These results were also validated by qPCR using the same oligos (Supplementary Fig. 1c, online resource) and different, qPCR-optimized ones (Fig. 1c, d, Supplementary Table 2, online resource). Moreover, we were able to amplify whole-length *TIR-MAPT* cDNA from starting codon to intron 12 using specific primers (TauNt and TauD, Supplementary Table 2, online resource) in SH-SY5Y cells. This cDNA would correspond to *TIR-MAPT* with three repeats and no inserts (TIR-T30).

More importantly, *TIR-MAPT* polyA positive mRNA was found in both hippocampus and frontal cortex in human samples (Fig. 1e). Remarkably, *TIR-MAPT* expression levels in human brain were more than 100-fold the expression found in SH-SY5Y, suggesting a higher relevance of this RNA species in human brains than in cultured cells.

Additionally, RNA-seq data from 363 samples from 3 different brain regions of 180 healthy donors were used to confirm the existence of *TIR-MAPT* transcripts (Supplementary Table 3, online resource). *TIR-MAPT* gene mature mRNA containing the first part of intron 12 up until the canonical stop site was expressed in the majority of samples (Fig. 1f) supporting our previous findings in a big cohort of human samples. Gene expression of *MAPT* mature mRNA was compared with *TIR-MAPT* (Fig. 1g). Even though *TIR-MAPT* is expressed at lower levels, the great number of samples expressing this intron-retaining species suggests a relevant function in human brain. Among the RNA species containing intron 12, some of the reads would correspond to a putative Tau protein without inserts, and with 3 tandem repeats (T30), but substituting the 3′-terminal of the RNA for the sequence of intron 12 until the first stop codon; what we have named TIR-T30. The putative translation of this RNA species into a protein has been named W-T30, due to the presence of two tryptophan residues (W) in the sequence of intron 12 translation (Supplementary Fig. 2, online resource). This RNA species would be equivalent to that found in SH-SY5Y cells.

### W-Tau expression in human brain

With the aim of determining whether this new Tau isoform is present in human brain as a protein, we obtained a specific antibody against the unique 18 amino acid sequence of W-Tau peptide (KKVKGVGWVGCCPWVYGH; W-Tau antibody), corresponding to the translation of intron 12 until the canonical stop codon. To confirm the specificity of this antibody, we cloned *TIR-MAPT* cDNA obtained from SH-SY5Y (TIR-T30, which would correspond to W-T30 as a protein) into the expression vector pSG5 (216201, Agilent technologies) and used it to overexpress this isoform in HEK293T cells. We demonstrated that W-T30 could be specifically immunoprecipitated with both W-Tau (Abyntek) or Total Tau (NOVUSBIO, NB100-82247) antibodies (Fig. 2a) and then that W-Tau antibody only precipitates W-Tau without any background on control untreated cells (Fig. 2b). In both cases, the immunoprecipitated protein was confirmed to be Tau by detecting it by Western blot with Tau 7.51 antibody.

To confirm this specificity and contrast it with other Tau isoforms, we further modified the eukaryotic vector pSG5 to include exon 10 corresponding to the fourth tandem repeat of the microtubule-binding domain and the two Tau inserts (thus obtaining W-T42). Additionally, asparagine-endopeptidase-truncated Tau corresponding isoforms were also cloned into pSG5 expression vector (ET: Endopeptidase truncated; ET-T42 and ET-T30); mimicking the cut of endopeptidase by including a stop codon after the one that encodes asparagine 368. We used this vector encoding W-Tau, ET-Tau and the full-length equivalents (T42 and T30) to overexpress these proteins on HEK293T cells, together with control cells transfected with the empty pSG5 vector. We observed that, while total Tau antibodies (such as Tau 7.51) recognize all these isoforms, W-Tau antibody only shows signal for W-Tau isoforms, without any background on other species (Fig. 2c).

Once the specificity of the antibody was confirmed, we probed human hippocampal and frontal lateral cortex samples with it, confirming the existence of this novel Tau isoform as a protein in human brain (Fig. 2d, e). In the first image, we show an example of a human subject that exhibits a predominant band close to 52 KDa that may correspond to a 4R form of W-Tau (Fig. 2d, 50 kDa band). Also, a smaller band at 38 KDa suggest the presence of W-T30 isoform in the hippocampal sample. Moreover, there is another band around 31 KDa that is recognized by W-Tau antibody, that might be explained as a proteolytic cleavage of the above bands, although further studies will be necessary to unravel the identity of this band. Then, we studied W-Tau expression in another three human subjects by comparing hippocampal and frontal lateral cortex samples (Fig. 2e). We could observe a predominant expression of 52 KDa 4R W-Tau bands, especially in frontal lateral cortex whose levels vary among individuals, and the 31 KDa band that may correspond to the truncated W-Tau form. All these results together indicate that intron 12 retaining Tau isoforms are expressed in human brain, of which two of them show similar molecular weights to those of W-T42 and W-T30 in cells upon overexpression.

### W-Tau phosphorylation

The pSG5 vectors encoding the different isoforms were employed to induce overexpression of these isoforms, to test W-Tau properties and compare them to those of full-length isoforms (T42 and T30) and those of asparagine-endopeptidase-truncated isoforms (ET-42 and ET30) (Fig. 3a). First, we studied the phosphorylation of W-Tau isoform, comparing it with the phosphorylation found in other Tau isoforms, expressed in HEK293T cells. We expected differential detections according to the epitopes that remain despite truncation and those that are lost because of it (Fig. 3a). Indeed, phosphorylation of AT8 sites Ser202/Thr205 and AT180 (phospho-Thr231) still occurred in truncated isoforms (Fig. 3b, c). However, as expected, PHF1 phosphorylation sites Ser396/Ser404 were not observed in truncated isoforms because these sequences are not present in those species. Also, some of the modifiable residues remained in dephosphorylated state, as shown by Tau 1 antibody label (dephospho- Ser195, 198, 199 and 202) (Fig. 3b, c) and negative staining for phospho-Thr212 and phospho-Ser214 (AT100) (data not shown).

These results point out a similar post-translational processing of W-Tau when compared to other Tau isoforms, at least regarding its phosphorylation on the mentioned epitopes.

### W-Tau microtubule stabilization and binding capacity

Then, we analyzed the effect of W-Tau on the polymerization of mouse brain microtubules in vitro by means of electron microscopy in the presence or absence of W-Tau, showing a higher proportion of microtubules in the presence of W-Tau (W-T42) (Supplementary Fig. 3a, online resource).

In addition, we analyzed the capacity of T42, W-T42, and ET-T42 purified from Tau-expressing bacteria (Supplementary Fig. 3b, online resource) to bind to microtubules purified from mouse brain, separating microtubule-bound and free Tau by centrifugation and characterizing Tau enrichment with respect to tubulin in microtubule-bound fraction by Western blot with Tau 5 antibody. As expected, all the tested isoforms were able to bind to microtubules (Supplementary Fig. 3b, c, online resource), since all of them contain microtubule-binding repeats. Of note, W-T42 showed a high affinity to bind microtubules compared to other isoforms harboring four repeats and two inserts (Supplementary Fig. 3c, online resource). In accordance with previously published results [63], ET-T42 showed lower affinity to bind microtubules than full-length Tau.

### W-Tau aggregation capacity in cultured cells

We compared the aggregation tendency of different Tau isoforms when overexpressed in HEK293T cells, by quantification of Tau in Sarkosyl soluble versus insoluble protein fractions, with the insoluble fraction including Tau aggregates (Fig. 4a). With respect to full-length T42 aggregation capacity, we could observe that a similar soluble/aggregated protein ratio was found in full-length Tau isoforms. However, in truncated Tau isoforms, the ratio was different in W-Tau isoforms, with a higher proportion of soluble protein when compared to both full-length and ET-Tau isoforms, being significatively higher for W-T30 with respect to its correspondent full-length T30 (Fig. 3b, c). These results were further confirmed by means of a similar assay that tested solubility with Triton X-100 (Supplementary Fig. 4a, online resource). A similar analysis could be performed with the signal obtained from W-Tau antibody (Supplementary Fig. 4b, c, online resource).

### In vitro aggregation of W-Tau

In addition, we carried out the analysis of the formation of protein aggregates by in vitro incubation of purified T42, W-T42 and ET-T42 with heparin, observing a reduced proportion of Tau aggregates when W-T42 was tested, compared to the rest of the isoforms (Fig. 4d), as determined by densitometry of electrophoretically fractionated protein bands present upon centrifugation in supernatant (non-aggregated protein) versus those present in pellet (aggregated protein) (Fig. 4e). These results support that W-Tau displays diminished aggregation tendency.

### Potential mechanism explaining intron 12 inclusion

We performed an in silico study, along with a bibliographic search, of the potential splicing factors involved in the process. Results obtained with ESEFinder 3.0 indicate the presence of a high-scored SRSF6 splicing binding site in the canonical *MAPT* sequence, close to the beginning of Exon 13 (Supplementary Fig. 5, online resource). This site is not present in the *TIR-MAPT* sequence. However, in its place, ESEFinder 3.0 indicates the appearance of a high-scored SRSF2 (also known as SC35) splicing binding site in intron 12, in contrast to *MAPT* sequence splicing out intron 12 (Supplementary Fig. 5, online resource). We re-analyzed *MAPT* exon 12–exon 13 and exon 12–intron 12 junction sequences using SRSF2 previously described binding sites [11, 38]. Surprisingly, we found that exon 12–intron 12 junction sequence, AGGTAAAG, is extremely similar to a specific SRSF2 binding site AGGTRAG (R for any Purine, A or G) previously described by Masaki et al. [38]. Both analyses suggested that SRSF2 may bind to *TIR-MAPT* sequence at the exon 12–intron 12 junction and/or at the beginning of intron 12 pointing to SRSF2 as a potential responsible for the regulation of this process. This evidence may be further supported by previous well-established evidence that SRSF2 is also involved in Tau exon 10 splicing [29], pointing out a regulatory function of this splicing factor on Tau alternative splicing that may be linked to the intron retention described in this work.

Additionally, previous work of our group suggests that the inhibition of GSK3-mediated SRSF2 phosphorylation increases the inclusion of exon 10 [29]. Thus, we tested the effect of the inhibition of SRSF2 phosphorylation carried out by the constitutively active GSK3 kinase [29] on the retention of *MAPT* intron 12. We detected increased *TIR-MAPT* cytoplasmic mature RNA levels in SK-N-MC cells in the presence of the GSK3β inhibitor AR-014418 [7], compared to both untreated and amyloid β treated cells (Fig. 5a). When we analyzed W-Tau protein levels, we could observe that SB216763, other GSK3β inhibitor also results in increased W-Tau levels, although this increase is more relevant for AR-014418 (Fig. 5b). These preliminary data indicate that SRSF2 may be involved in this process via GSK3β regulation (Fig. 5c).

Finally, it is worth noting that the presence of beta amyloid peptide, that could stimulate GSK3 [28], did not result in a change in the level of neither *TIR-MAPT* nor W-Tau (Fig. 5a, b), probably because GSK3 is constitutively active and, therefore, amyloid-treated cells behave as untreated control cells containing already SC35 in its phosphorylated form. However, as previously described, amyloid β did result in a significant increase of total *MAPT* RNA (Fig. 5a).

### Expression of *TIR-MAPT* RNA in the brain of AD patients.

**Fig. 6:**
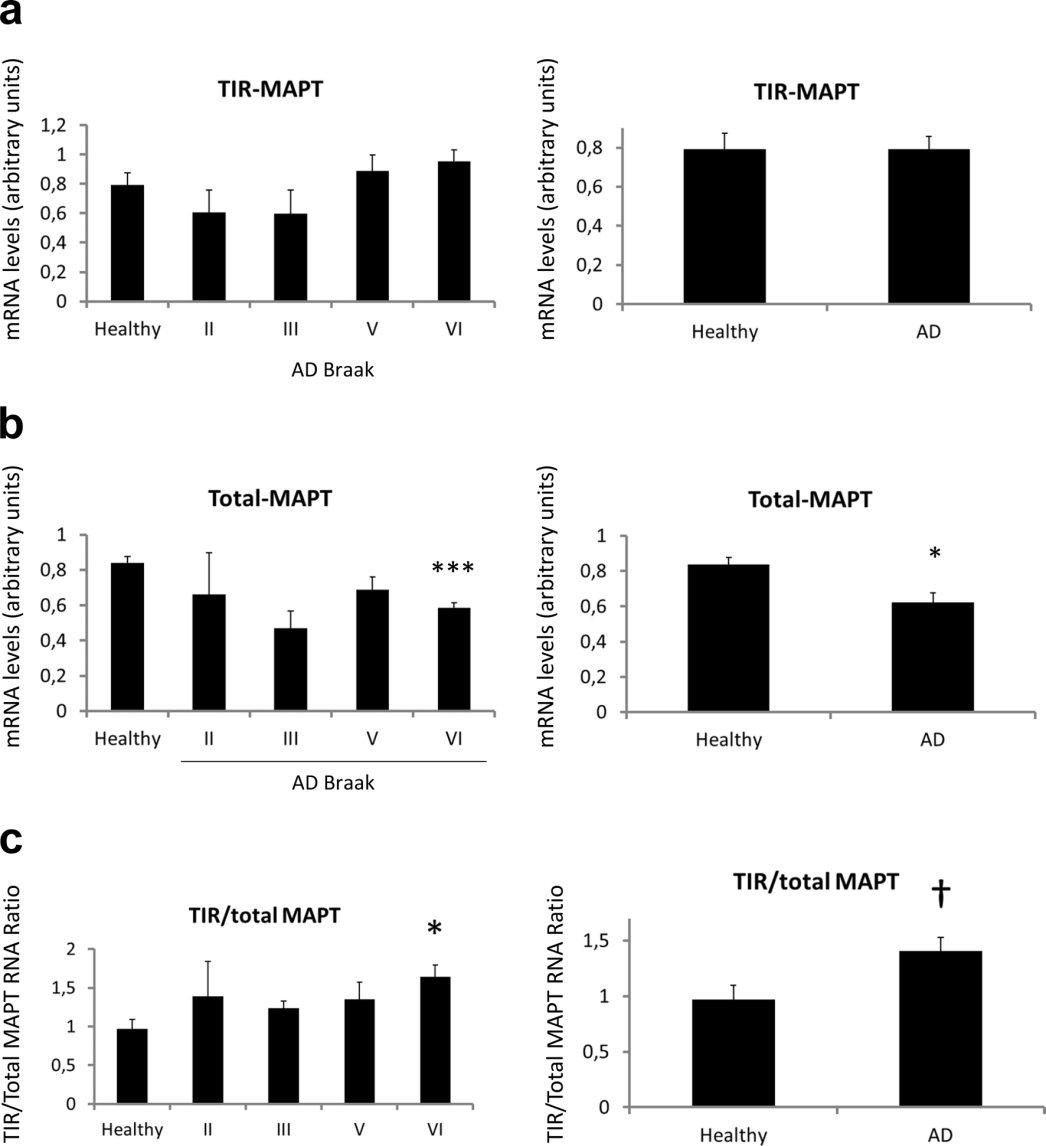
*TIR-MAPT* RNA expression in the brains of AD patients. **a** Measurement of *TIR-MAPT*, **b** total *MAPT* and **c** *TIR-MAPT*/total *MAPT* ratio of RNA levels by RT-qPCR of non-demented (*n* = 8) and AD hippocampal samples (*n* = 16) classified according to their Braak stage (Braak II *n* = 3, Braak III *n* = 2, Braak V *n* = 7 and Braak VI *n* = 4), and healthy vs. AD patients. Graphs show means and SE. One-way ANOVA and Dunnett’s multiple comparisons test were performed and statistical significance of each group with respect to non-demented control individuals was given. For comparisons between non-demented individuals and AD patients as groups, *p* values were calculated using a *T* test (*p ≤ 0.05; ***p ≤ 0.001, ^†^*p* = 0,078)

We studied *TIR-MAPT* mRNA levels in a cohort of hippocampal brain samples from non-demented individuals and AD patients classified according to their Braak stage (see Supplementary Table 1, online resource). The study of absolute levels of *TIR-MAPT* mRNA revealed that it slightly decreases in the first stages of the disease (Braak II and III) but increases in the advanced stages (Braak V and VI), although no significant differences were found (Fig. 6a). Total *MAPT* mRNA levels were similarly studied, finding in this case decreased expression in AD patients with respect to healthy individuals, more significantly in Braak stage VI (Fig. 6b). These data correlate with what we previously described in a human RNA-seq analysis [23]. Finally, when we studied *TIR-MAPT*/total *MAPT* ratio we could observe a significant increase in Braak VI samples as well as a tendency towards an increased ratio in AD patients with respect to non-demented individuals (Fig. 6c).

### W-Tau presence in patients’ brains

**Fig. 7:**
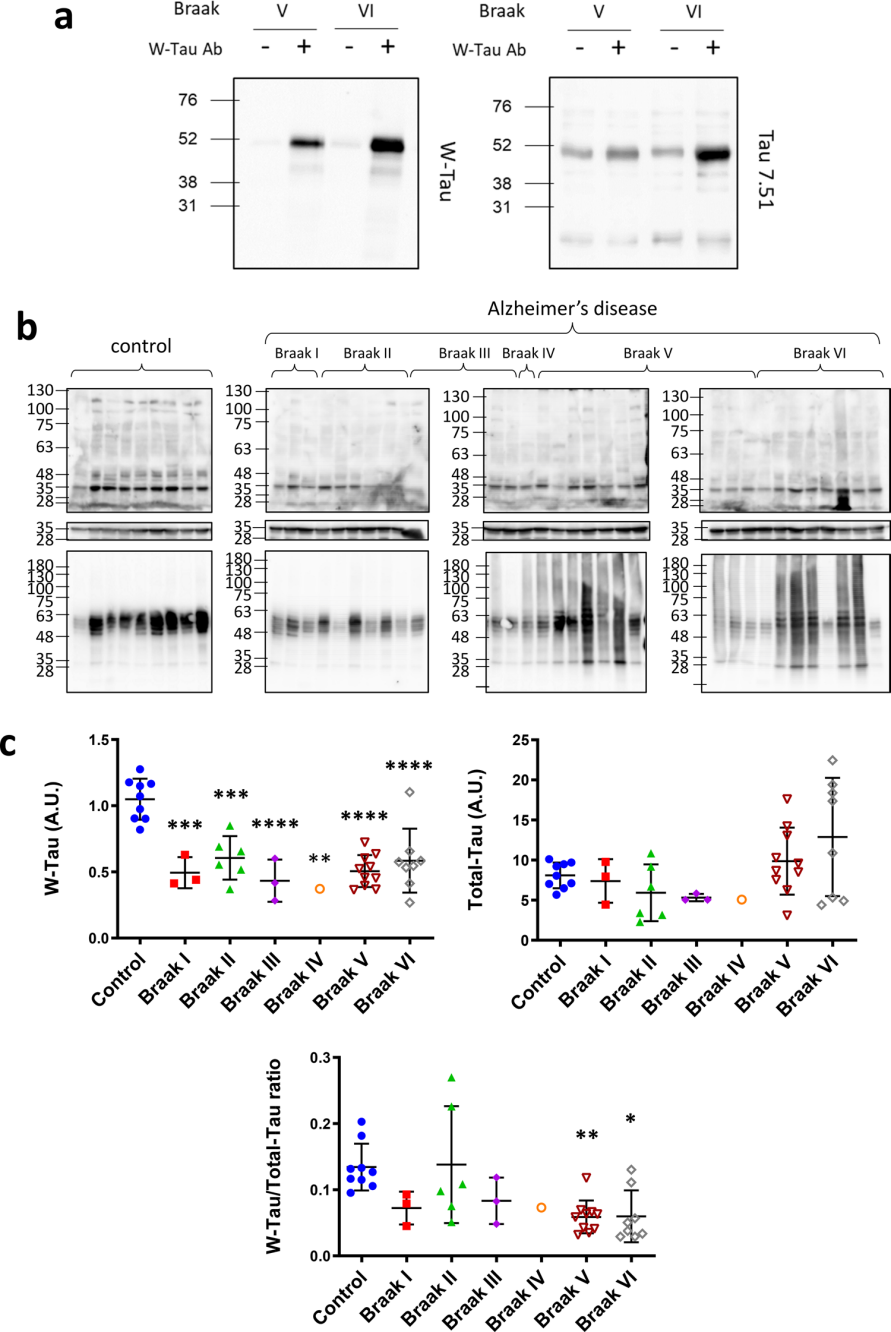
Tau protein determination in AD patients’ brain. **a** Immunodetection of the presence of W-Tau isoforms using W-Tau antibody in two different frontal lateral cortex brain extracts derived from AD patients’ brains (Braak V and VI, respectively). The immunoprecipitate performed with W-Tau antibody was characterized by Westernblotting. Left panel shows the blot developed using W-Tau antibody; right panel using Tau 7.51 antibody. **b** Western blot analysis of the levels of W-Tau and total Tau in frontal lateral cortical samples of non-demented (*n* = 9) and AD patients classified according to their Braak stage (Braak I = 3; Braak II *n* = 6, Braak III *n* = 3, Braak IV *n* = 1, and Braak V *n* = 10, Braak VI *n* = 8). **c** Quantification of W-Tau and total Tau protein levels as well as W-Tau/total Tau ratio of each group. One-way ANOVA and Dunnett’s multiple comparisons test were performed and statistical significance of each group with respect to non-demented control individuals was given (*p ≤ 0.05; **p ≤ 0.01; ***p ≤ 0.001; ****p ≤ 0.0001). *A.U.* arbitrary units

Finally, in accordance to what was found for control human brains (Fig. 2d, e), we confirmed that W-Tau antibody was able to specifically immunoprecipitate W-Tau protein from human brain in extracts from AD patients (Braak stages V and VI) that could be identified as Tau again with a total Tau antibody (Tau 7.51) (Fig. 7a).

Once this was confirmed, we analyzed the levels of W-Tau protein in the cortex of healthy individuals and AD patients classified according to their Braak stage (Fig. 7b). We could observe that W-Tau is expressed in both healthy and AD individuals. The analysis of both W-Tau bands showed a strong diminished expression in all Braak stages with respect to non-demented individuals (Fig. 7c). Accordingly, the quantification of each W-Tau band separately also yielded significant differences in all Braak stages with respect to non-demented individuals (Supplementary Fig. 6a, online resource). On the other hand, the expression of total Tau seems to increase at later stages of the disease, but we could not detect significant differences among groups when individuals are separated by Braak stages in our samples (Fig. 7c). However, the ratio of W-Tau with respect to total Tau expression reveals an enrichment of W-Tau in advanced stages of the disease (Braak V and VI) (Fig. 7c).

Additionally, we could also obtain hippocampal samples of some of these individuals, and we studied W-Tau expression (Supplementary Fig. 6b, online resource). In such conditions, similar to what we previously observed in Fig. 2d, e, we mainly detect 31 KDa (W-Tau truncated) and 50 KDa (W-T42) bands and, to a lesser extent, 38 KDa (W-T30) band. We could observe a trend towards diminished W-Tau expression in AD patients in the tree bands being significant in advanced stages of the disease for the 38 KDa (W-T30) band as well as in W-Tau/total Tau ratio (Supplementary Fig. 6c, d, online resource).

These data together indicate an inverse correlation between W-Tau levels and disease progression that opposes total Tau levels behavior, strongly suggesting a relevant role of this new intron-retaining isoform in healthy individuals, whose loss would be related to the progression of the disease. Taken RNA and protein data together (Figs. 6, 7) and considering the more marked presence of Tau aggregates in Total Tau but not in W-Tau, the changes on the amount of W-Tau in AD with respect to total Tau may be more related to differences on the protein turnover, rather than to their synthesis.

## Discussion

Although recent evidence point out that a large proportion of predicted alternative transcripts might not be translated into proteins [55], *MAPT* gene has been proven to be genuinely subjected to alternative splicing [1, 55]. Also, *MAPT* contains a cryptic exonic sequence codifying for the protein saitohin nested in the Tau gene within intron 9 [14]. Thus, the removal of RNA sequences in Tau RNA is very usual, but the translation of intronic sequences could also take place. Curiously, it has been suggested that the region of “big” Tau corresponding to exon 4a arose, evolutionary, from an intron from another protein [20].

Tau alternative splicing is also regulated by several features such as development, aging, disease or brain region [8, 54, 56, 58]. Tau isoforms lacking exons 2, 3 and 10, can be mainly found at early developmental stages [2, 56] and alternative splicing of exon 10 has been related to some tauopathies [10, 50].

Recently, a toxic fragment containing residues 1–368 has been reported [63]. Since residue 368 coincides with the end of exon 12, in this work we have studied whether a failure in the removal of intron 12 could give rise to a novel Tau isoform. We found that intron 12 retention can take place, in low proportions in non-demented human control as well as AD patients’ brains. The failure of intron 12 removal results in the appearance of a Tau isoform containing 18 additional residues after exon 12. Strikingly, this new isoform is significantly reduced in AD, especially in the advanced stages, while total Tau is accumulated. It is worth emphasizing that this isoform is human-specific, since other species’ Tau orthologues do not contain the polyadenylation canonical site that allows intron retention to give rise to a truncated protein. This is especially noteworthy considering that Alzheimer’s disease is a human pathology that is not well reproduced in animal models.

A possible mechanism explaining intron 12 retention is that trans-acting splicing factors could be modified, for example by phosphorylation, and that the modified factor could change its function. Moreover, modification of intron splicing has been previously reported for SR-proteins [40] and, specifically, for SRSF2 (SC35) which, when phosphorylated by GSK3 kinase, favors the expression of Tau 3R (lacking exon 10) [29]. Additionally, it has been described that the absence of SRSF2 favors exon 10 inclusion increasing Tau 4R species [12]. Notably, intron splicing events have been previously reported for SRSF2 and other member of the SR-proteins family [40]. In this work, we found that GSK3 inhibition also induces the retention of intron 12, presumably by the inhibition of SRSF2 function, inducing the expression of *TIR-MAPT* mRNA and protein, while total Tau levels remain unmodified. SRSF2 could play a role in this process, since there are two open recognition sites for this factor in *TIR-MAPT* sequence, one of them located at the exon 12–intron 12 junction [11, 38]. Of note, W-Tau levels did not change in amyloid-treated cultured cells, even though amyloid beta peptide has been reported to stimulate GSK3 [12, 28]. This may be due to GSK3 being constitutively active and, consequently, amyloid-mediated activation does not imply further SRSF2 phosphorylation when comparison to untreated cells.

The novel Tau fragment described in this work is very similar to the previously reported fragment generated by cleavage of AEP [63]. However, contrary to what was expected, the isoforms truncated by intron retention exhibit a potentially protective behavior, with decreased aggregation capacity but conservation of microtubule assembly capacity. This novel Tau isoforms contain a unique 18-aminoacid extra peptide, including the sequence GVGWVG, which could be similar in nature to that of LYIWVQ, a recently described inhibitor of Tau and Aβ aggregation [27] (Supplementary Fig. 7, online resource). Thus, we suggest that the decreased aggregation capacity of W-Tau could be related to the presence of this extra peptide. Moreover, W-Tau lacks the 12 amino acids after the fourth repeat, present in exon 13, which are found in the core of Tau filaments isolated from the brain of patients suffering some tauopathies, including Alzheimer’s disease [62]. It is also worth noting that the specific W-Tau 18 amino acid sequence contains two adjacent cysteines (KKVKGVGWVGCCPWVYGH), whose oxidation to cystine can mediate the formation of a strained eight-member ring [47], a conformation that would substantially differ of that of other Tau isoforms and that has been proposed to be able to affect protein function and conformation stability [33, 39].

As for the relevance of this novel truncated isoform in AD’s development and progression, similar *TIR-MAPT* mRNA levels were found in non-demented control subjects and AD patients’ samples. However, on a protein level, we have detected higher levels of W-Tau protein in healthy individuals compared to AD patients. Discordant RNA and protein levels for Tau in AD have been previously described [23]. We hypothesize that, in this case, it may be related to a relatively faster turnover for W-Tau isoforms, maybe linked to a different conformation rising from its different sequence. These data also showed a clearly less aggregative pattern on W-Tau isoforms when compared to total Tau (Fig. 7), suggesting that this is a less aggregation-prone Tau isoform indeed.

Finally, therapeutic correction of aberrant splicing that could take place in Alzheimer’s disease has been suggested for some nuclear RNAs [4, 31]. Given the potential role of this new Tau truncated isoform on a possible AD therapy, we will look for future therapeutic strategies based on an increase of this less aggregation-prone Tau isoform or the prevention of its decrease.

## Supplementary Information

Below is the link to the electronic supplementary material.

Supplementary file1 (PDF 833 kb)
