# Supplementary material for: A new non-aggregative splicing isoform of human Tau is decreased in Alzheimer’s disease
Source: Acta Neuropathol. 2021 May 2;142(1):159–77. doi: 10.1007/s00401-021-02317-z (PMC8217066; doi:10.1007/s00401-021-02317-z)
Supplement: Supplementary file 1 — Supplementary file1 (PDF 833 kb) [file 401_2021_2317_MOESM1_ESM.pdf]

Supplementary Figure 1 Semiquantitative and quantitative determination of W-Tau in SH-SY5Y. a. Schematic representation of the hybridization sites of the primers (detailed in **Supplementary Table 2**) designed for semi-quantitative PCR in *MAPT* sequence. **b.** Representative images of agarose gels showing semi-quantitative PCR results using total or cytoplasmic-enriched mRNA of SH-SY5Y cells. Results showed the existence of RNA species from exon 12 to intron 12 (PCR 1 and 2) and from exon 11 to intron 12 where intron 11 was spliced out (PCR 3 and 4). On the contrary, RNA species from exon 10 to intron 12 where introns 10 and 11 were spliced out were not found in cytoplasmic mature enriched fraction (PCR 7 and 8). RNA species where intron 12 was spliced out were found in all fractions (PCR 10, 11 and 12). Controls of the addition (RT+) or no addition of retrotranscriptase (RT-) were included. PCR 13 amplifying GAPDH transcript was used as control. Detailed information of all semi-quantitative PCR combinations and amplicon sizes is provided in Supplementary Table 5. **c**. Quantitative RT-PCR analysis of the same analysis shown in **b**.

Supplementary Figure 2 Comparison of predicted translation of *MAPT* canonical transcript and intron 12-retaining *MAPT* transcript. a. Tau protein sequence for the isoform containing 3 tandem repeats without inserts, compared to the predicted translation of intron 12-retaining *MAPT* RNA sequences with 3 tandem repeats and without inserts. The highlighted fragments correspond to the exons and intron depicted in Fig. 1a. The difference between both sequences is marked in bold letters. **b.** Nucleotide cDNA sequence of *TIR-MAPT* and corresponding amino acid sequence of W-Tau with two inserts and 4 repeats (W-T42). The part of the sequence highlighted in orange indicates the *TIR-MAPT* or W-Tau specific sequence corresponding to intron 12 retention up to the stop codon TAA.

Supplementary Figure 3 Microtubule binding capacity of W-Tau. **a**. Electronic microscopy images comparing microtubule polymerization in the absence or presence of W-T42 isoform and corresponding quantification of microtubules per field. Seven fields were analyzed for control lacking W-T42 and 13 fields were analyzed for samples containing W-T42. Graph show mean and SEM (**** $p \leq 0.0001$). **b**. Representative Western blot images of T42, W-T42 and ET-T42 purified from bacteria, as were used for microtubule-binding experiments. **c**. Western blot analysis of the microtubule-bound Tau after copolymerization of different human Tau isoforms with mouse brain microtubules detected with an anti-human Tau antibody (HT7). Quantification of Tau/tubulin ratio of each Tau isoform with respect to the full-length isoform (T42). Graph show means and SEM of three independent experiments for 4R isoforms. One-way ANOVA for multiple comparisons followed by a Kruskal-Wallis test was performed and statistical significance of each isoform with respect to T42 was given. Results were not significative.

Supplementary Figure 4 W-Tau aggregation capacity. a. Representative Western blot of the presence of Tau in 1% Triton X-100-soluble and insoluble cell fractions of HEK293T cells overexpressing different Tau isoforms (T42, T30, W-T42, W-T30, ET-42 and ET-T30), detected with 7.51 antibody. **b**. Representative Western blot of the presence of W-Tau in 1% sarkosyl-soluble and insoluble cell fractions of HEK293T cell overexpressing different Tau isoforms (T42,T30, W-T42, W-T30, ET-42 and ET-T30) detected with W-Tau antibody. **c**. Average of the quantification of the signal obtained with W-Tau antibody in two independent experiments.

Supplementary Figure 5 Study of potential splicing factors involved in the exclusion or inclusion of intron 12. Literature searching and *in silico* study using ESEFinder 3.0 (http://exon.cshl.edu/ESE/) of the exon 12—exon 13 or exon 12—intron 12 boundary of human *MAPT* **a**. Sequence of exon 12—exon 13 boundary in *MAPT* RNA. Splicing factor binding site predicted by ESEFinder for SRSF6 is indicated. **b**. Sequence of exon 12—intron 12 human *MAPT* RNA, including the coding sequence expressing the extra peptide present in W-Tau. The exon 12—intron 12 junction is similar to the low-affinity SRSF2 binding sequence reported by Masaki et al, 2019 (AGGTAAAG), and ESEFinder predicts another SRSF2 binding site (GGATGCTG). **c.** Schematic representation of the splicing factors binding sites in full-length *MAPT* species versus *TIR-MAPT* species.

Supplementary Figure 6 W-Tau expression in human brain a. Quantification of W-Tau 38 KDa (W-T30) and 52 KDa band (W-T42) of the western blot shown in Fig. 7b of frontal lateral cortex samples of non-demented subjects (n=9) and AD patients classified according to their Braak stage (Braak I = 3; Braak II n=6, Braak III n=3, Braak IV n=1, and Braak V n=10, Braak VI n=8). **b.** Representative Western blot analysis of W-Tau and total-Tau (Tau5) expression in hippocampal brain samples of non-demented (n=9) and AD patients classified according to their Braak stage (Braak II n=3, Braak III n=3, Braak V n=4 and Braak VI n=4). **c.** Quantification of each W-Tau band 31 KDa (W-Tau truncated), 38 KDa (W-T30) or 52 KDa (W-T42) in non-demented (control) individuals, early-mild AD (Braak II-III) or severe AD (Braak V-VI). **d**. Similar quantification of the expression of W-Tau bands with respect to total-Tau expression. One-way ANOVA and Dunnett's multiple comparisons test were performed and statistical significance of each group with respect to non-demented control individuals was given (* $p \leq 0.05$; ** $p \leq 0.01$; *** $p \leq 0.001$; **** $p \leq 0.0001$). A.U.: Arbitrary Units.

Supplementary Figure 7 Comparison of the amino acid sequence of the W-Tau peptide with those of different Tau-Aβ cross-seeding inhibitors. These inhibitors are described in Griner et al [26].

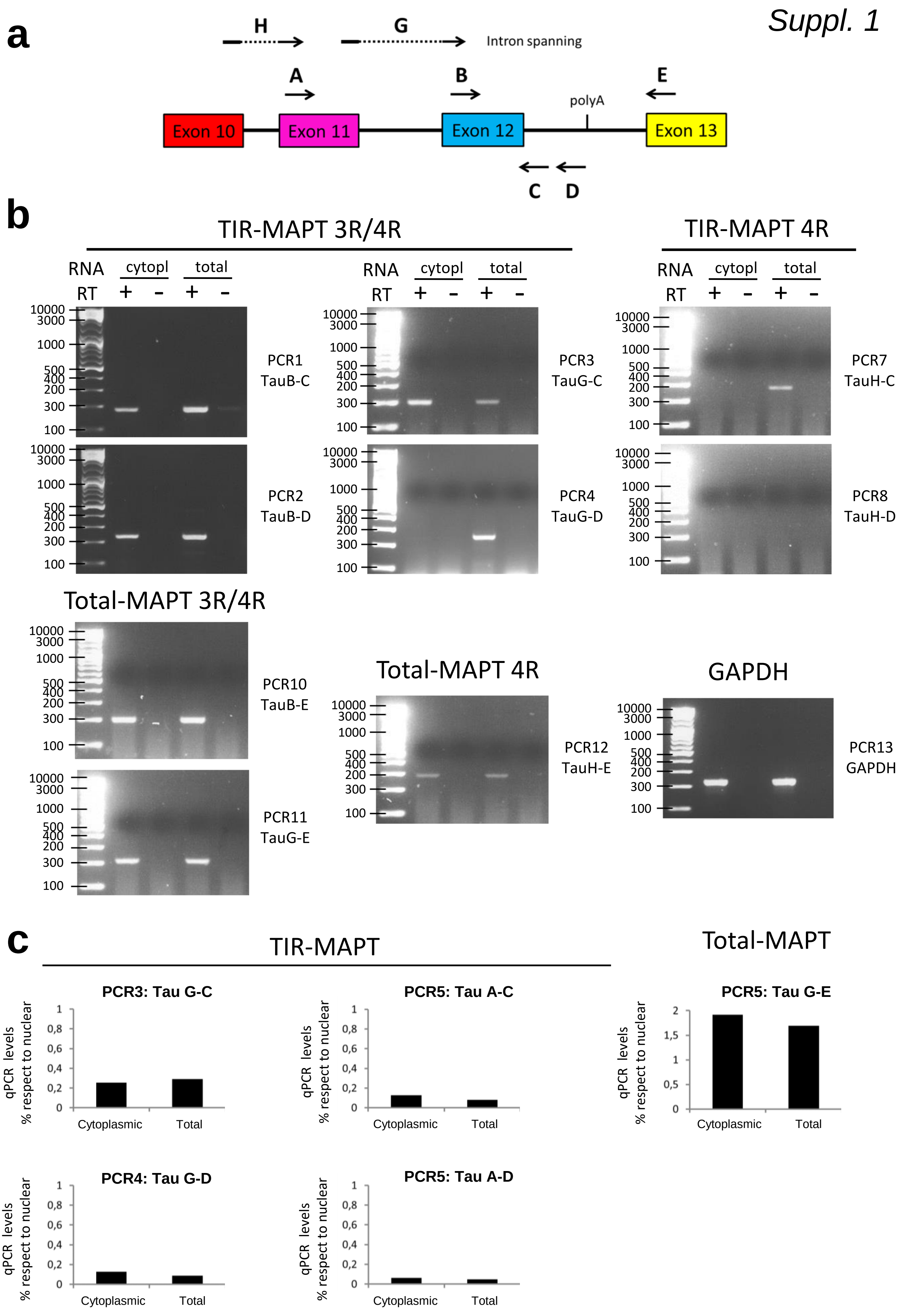

Suppl. 1
a
H
G
Intron spanning
A
B
E
polyA
Exon 10
Exon 11
Exon 12
Exon 13
C
D
b
TIR-MAPT 3R/4R
TIR-MAPT 4R
RNA cytopl total
RT + – + –
PCR1 TauB-C
PCR2 TauB-D
PCR3 TauG-C
PCR4 TauG-D
PCR7 TauH-C
PCR8 TauH-D
Total-MAPT 3R/4R
PCR10 TauB-E
PCR11 TauG-E
Total-MAPT 4R
PCR12 TauH-E
GAPDH
PCR13 GAPDH
10000 3000 1000 500 400 200 300 100
c
TIR-MAPT
Total-MAPT
PCR3: Tau G-C
PCR5: Tau A-C
PCR5: Tau G-E
PCR4: Tau G-D
PCR5: Tau A-D
qPCR levels % respect to nuclear
Cytoplasmic
Total

a

> Tau with no inserts and 3 microtubule-binding domains (T30)
MAEPRQEFEVMEDHAGTYGLGDRKDQGGYTMHQDQEGDTDAGLKAEEAGIGDTPSLEDE
AAGHVTQARMVSKSKDGTGSDDKKAKGADGKTKIATPRGAAPPGQKGQANATRIPAKTPP
APKTPPSSGEPPKSGDRSGYSSPGSPGTPGSRSRTPSLPTPPTREPKKVAVVRTPPKSPSSAKS
RLQTAPVPMPDLKNVKSKIGSTENLKHQPGGGKVQIVYKPVDLSKVTSKCGSLGNIHHKPG
GGQVEVKSEKLDFKDRVQSKIGSLDNITHVPGGGN**KKIETHKLTFRENAKAKTDHGAEIV**
YKSPVVSGDTSPRHLSNVSSTGSIDMVDSPQLATLADEVSASLAKQGL

>W-Tau with no inserts and 3 microtubule-binding domains (W-T30)
MAEPRQEFEVMEDHAGTYGLGDRKDQGGYTMHQDQEGDTDAGLKAEEAGIGDTPSLEDE
AAGHVTQARMVSKSKDGTGSDDKKAKGADGKTKIATPRGAAPPGQKGQANATRIPAKTPP
APKTPPSSGEPPKSGDRSGYSSPGSPGTPGSRSRTPSLPTPPTREPKKVAVVRTPPKSPSSAKS
RLQTAPVPMPDLKNVKSKIGSTENLKHQPGGGKVQIVYKPVDLSKVTSKCGSLGNIHHKPG
GGQVEVKSEKLDFKDRVQSKIGSLDNITHVPGGGN**KKVKGVGWVGCCPWVYGH**

b

>TIR-MAPT cDNA with 2 inserts and 4 repetitions:
ATGGCTGAGCCCCGCCAGGAGTTCGAAGTGATGGAAGATCACGCTGGGACGTACGGGTTG
GGGGACAGGAAAGATCAGGGGGGCTACACCATGCACCAAGACCAAGAGGGTGACACGGA
CGCTGGCCTGAAAGAATCTCCCCTGCAGACCCCCACTGAGGACGGATCTGAGGAACCGGG
CTCTGAAACCTCTGATGCTAAGAGCACTCCAACAGCGGAAGATGTGACAGCACCCTTAGTG
GATGAGGGAGCTCCCGGCAAGCAGGCTGCCGCGCAGCCCCACACGGAGATCCCAGAAGG
AACCACAGCTGAAGAAGCAGGCATTGGAGACACCCCCAGCCTGGAAGACGAAGCTGCTG
GTCACGTGACCCAAGCTCGCATGGTCAGTAAAAGCAAAGACGGGACTGGAAGCGATGACA
AAAAAGCCAAGGGGGCTGATGGTAAAACGAAGATCGCCACACCGCGGGGAGCAGCCCCT
CCAGGCCAGAAGGGCCAGGCCAACGCCACCAGGATTCCAGCAAAAACCCCGCCCGCTCCA
AAGACACCACCCAGCTCTGGTGAACCTCCAAAATCAGGGGATCGCAGCGGCTACAGCAGC
CCCGGCTCCCCAGGCACTCCCGGCAGCCGCTCCCGCACCCCGTCCCTTCCAACCCCACCCAC
CCGGGAGCCCAAGAAGGTGGCAGTGGTCCGTACTCCACCCAAGTCGCCGTCTTCCGCCAAG
AGCCGCCTGCAGACAGCCCCCGTGCCCATGCCAGACCTGAAGAATGTCAAGTCCAAGATCG
GCTCCACTGAGAACCTGAAGCACCAGCCGGGAGGCGGGAAGGTGCAGATAATTAATAAGA
AGCTGGATCTTAGCAACGTCCAGTCCAAGTGTGGCTCAAAGGATAATATCAAACACGTCCCG
GGAGGCGGCAGTGTGCAAATAGTCTACAAACCAGTTGACCTGAGCAAGGTGACCTCCAAGT
GTGGCTCATTAGGCAACATCCATCATAAACCAGGAGGTGGCCAGGTGGAAGTAAAATCTGA
GAAGCTTGACTTCAAGGACAGAGTCCAGTCGAAGATTGGGTCCCTGGACAATATCACCCAC
GTCCCTGGCGGAGGAAAT**AAAAAGGTAAAGGGGGTAGGGTGGGTTGGATGCTGCCCTT**
GGGTATATGGGCATTAA

>W-T42 protein sequence:
MAEPRQEFEVMEDHAGTYGLGDRKDQGGYTMHQDQEGDTDAGLKESPLQTPTEDGSEEPGSET
SDAKSTPTAEDVTAPLVDEGAPGKQAAAQPHTEIPEGTTAEEAGIGDTPSLEDEAAGHVTQARMVS
KSKDGTGSDDKKAKGADGKTKIATPRGAAPPGQKGQANATRIPAKTPPAPKTPPSSGEPPKSGDR
SGYSSPGSPGTPGSRSRTPSLPTPPTREPKKVAVVRTPPKSPSSAKSRLQTAPVPMPDLKNVKSKIGS
TENLKHQPGGGKVQIINKKLDLSNVQSKCGSKDNIKHVPGGGSVQIVYKPVDLSKVTSKCGSLGNI
HHKPGGGQVEVKSEKLDFKDRVQSKIGSLDNITHVPGGGN**KKVKGVGWVGCCPWVYGH**

a

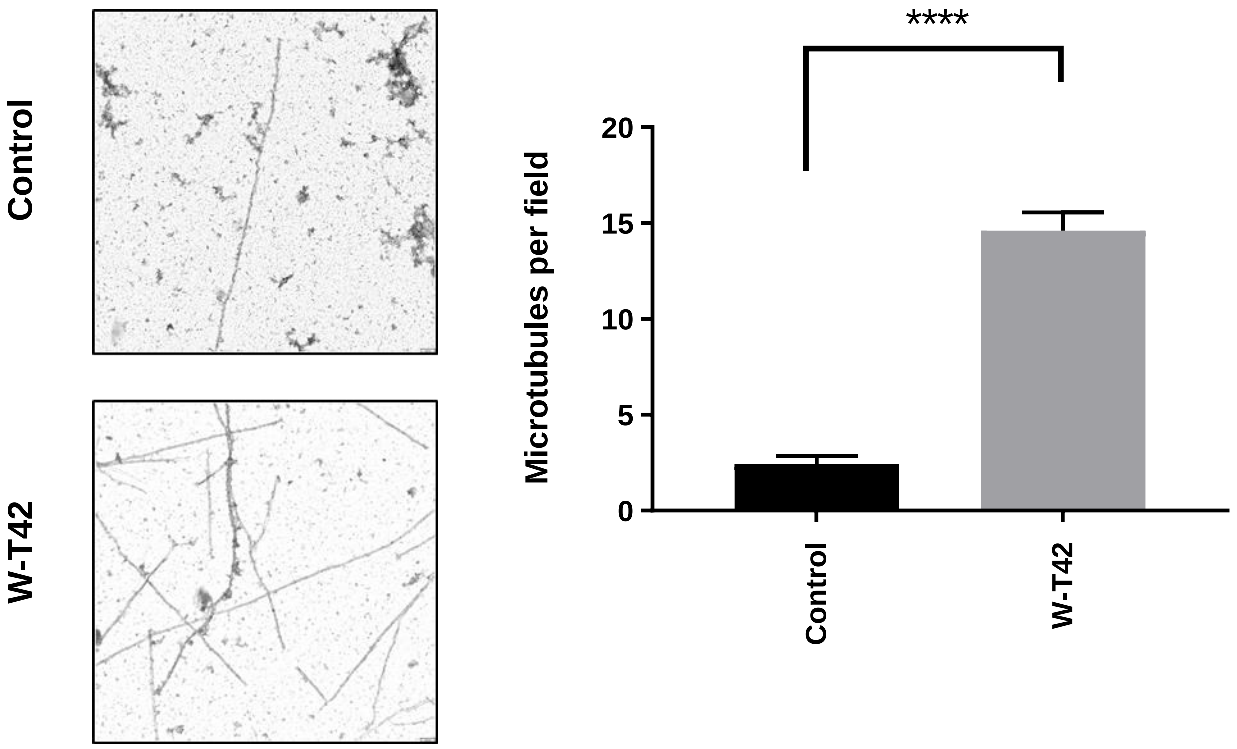
Control
W-T42
****
Microtubules per field
20
15
10
5
0
Control
W-T42

b

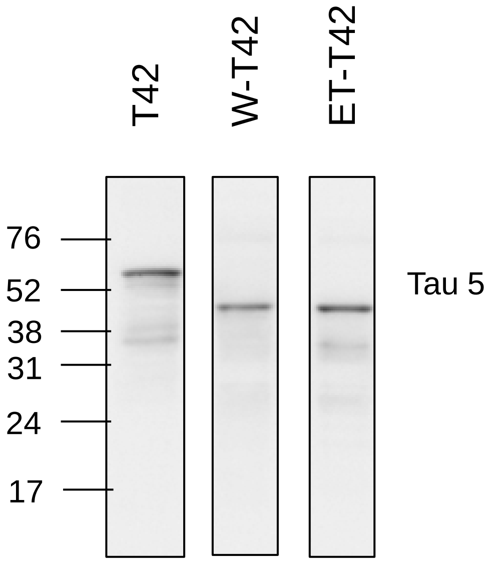
T42
W-T42
ET-T42
76
52
38
31
24
17
Tau 5

c

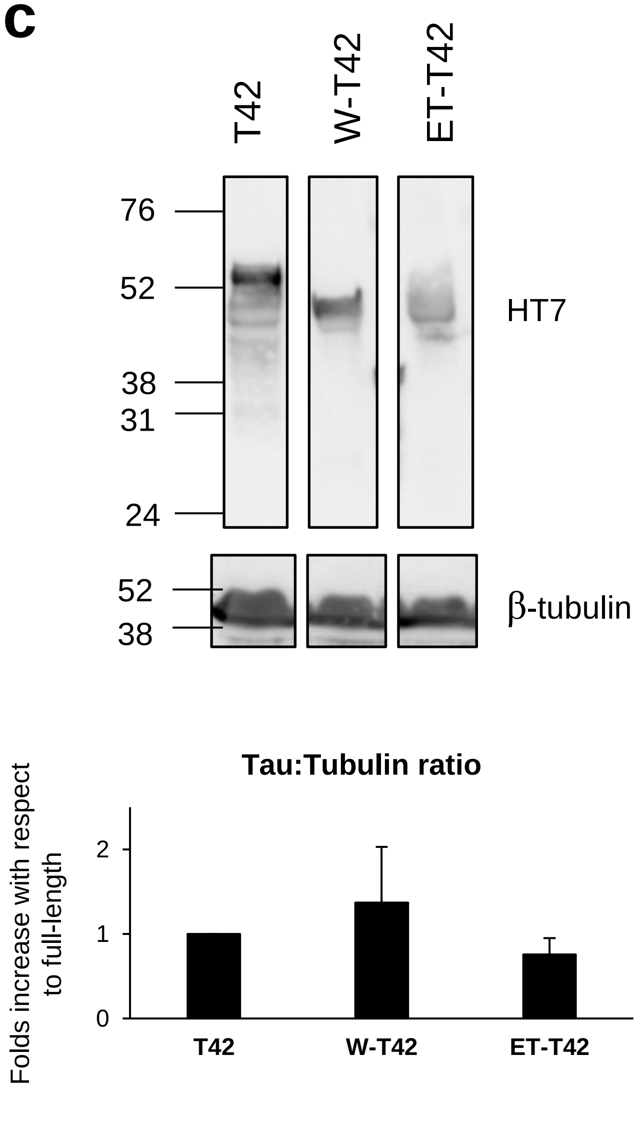
T42
W-T42
ET-T42
76
52
38
31
24
HT7
52
38
β-tubulin
Tau:Tubulin ratio
Folds increase with respect to full-length
2
1
0
T42
W-T42
ET-T42

Suppl. 4

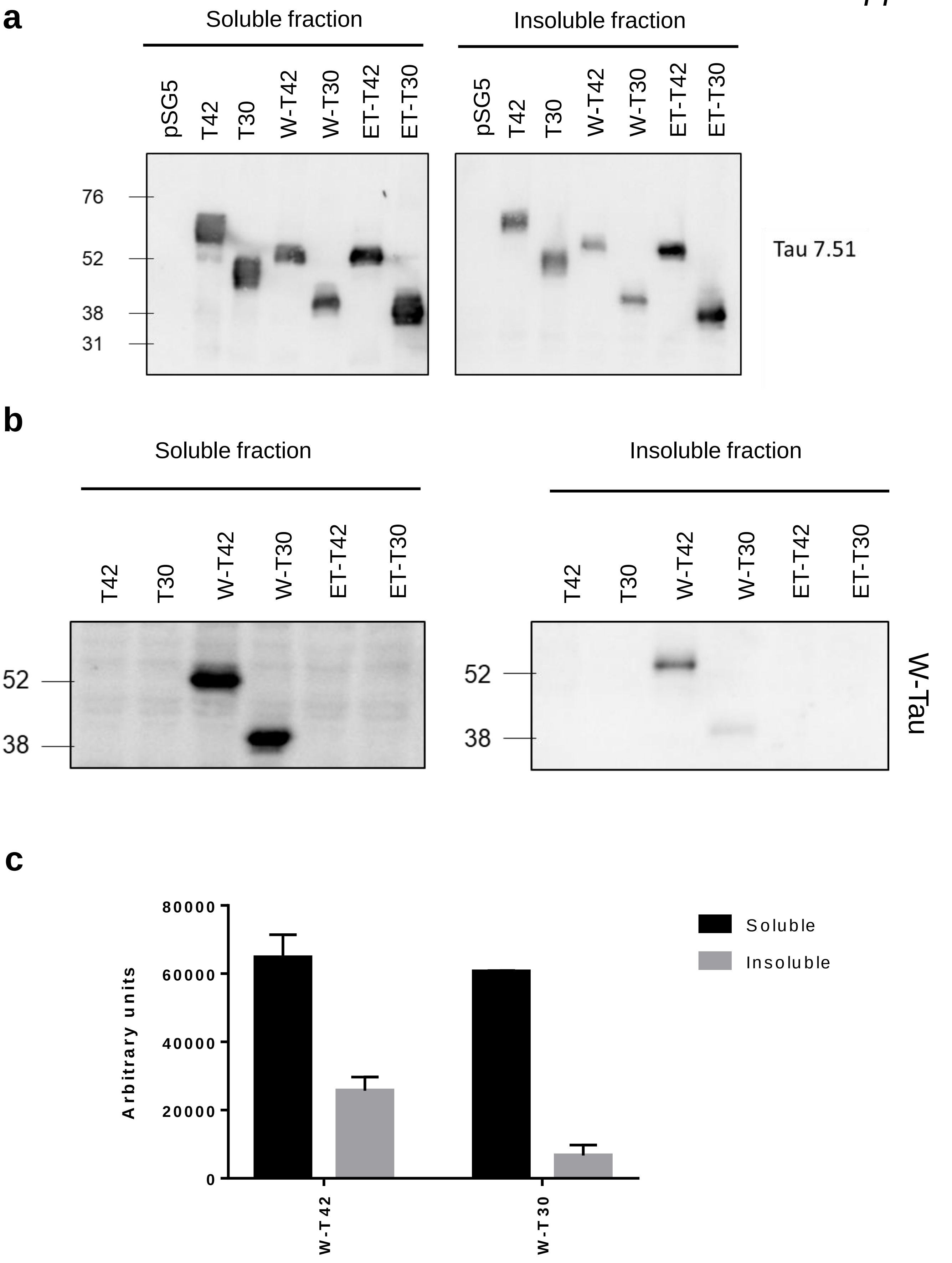

a
Soluble fraction
Insoluble fraction
pSG5
T42
T30
W-T42
W-T30
ET-T42
ET-T30
76
52
38
31
Tau 7.51
b
Soluble fraction
Insoluble fraction
52
38
W-Tau
c
80000
60000
40000
20000
0
Arbitrary units
Soluble
Insoluble
W-T42
W-T30

a Exon12-Exon13 (MAPT, NM_005910.5)

GAGGTGGCCAGGTGGAAGTAAAATCTGAGAAGCTTGACTTCAAG
GACAGAGTCCAGTCGAAGATTGGGTCCCTGGACAATATCACCCAC
GTCCCTGGCGGAGGAAATAAAAAGATTGAAACCCACAAGCTGAC
CTTCCGCGAGAACGCCAAAGCCAAGACAGACCACGGGGCGGAG
ATCGTGTACAAGTCGCCAGTGGTGTCTGGGGA**CACGTCT**CCACG
GCATCTCAGCAAT…

CACGTCT: ESE Finder Score value for SRSF6 binding site: 4.69.

b Exon12-Intron 12 (TIR-MAPT)

GAGGTGGCCAGGTGGAAGTAAAATCTGAGAAGCTTGACTTCAAG
GACAGAGTCCAGTCGAAGATTGGGTCCCTGGACAATATCACCCAC
GTCCCTGGCGGAGGAAATAAAA**AGGTAAAG**GGGGTAGGGTGG
GTT**GGATGCTG**CCCTTGGGTATATGGGCATTAATCAAGTTGAGTG
GACAAAGGCTGGTCCAGTTCCCAGAGGAGGAAAACAGAGGCTTC
TGTGTTGACTGGC…

AGGTAAAG: Similar to SRSF2 consensus site proposed in Masaki et al. 2019 (AGGTRAG).

GGATGCTG: ESE Finder Score value for SRSF2 binding site: 4.31.

c

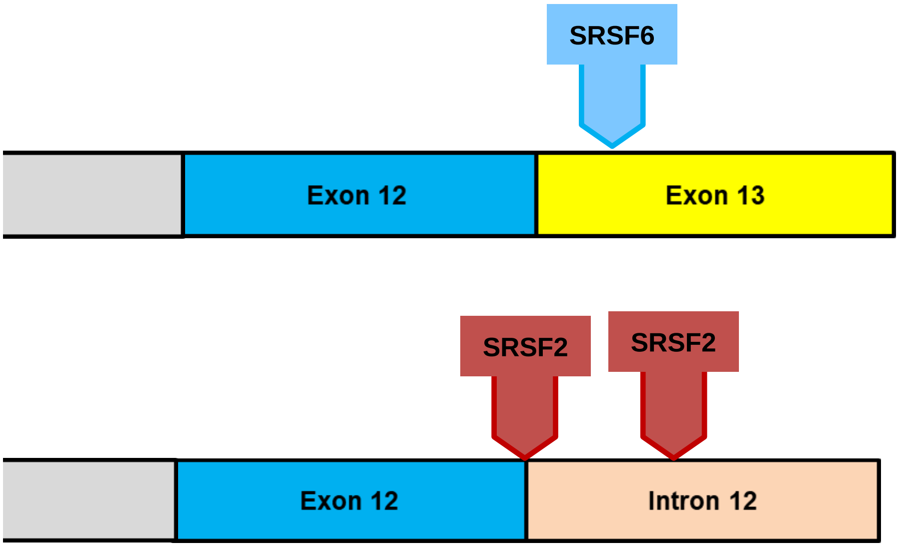

Suppl. 6

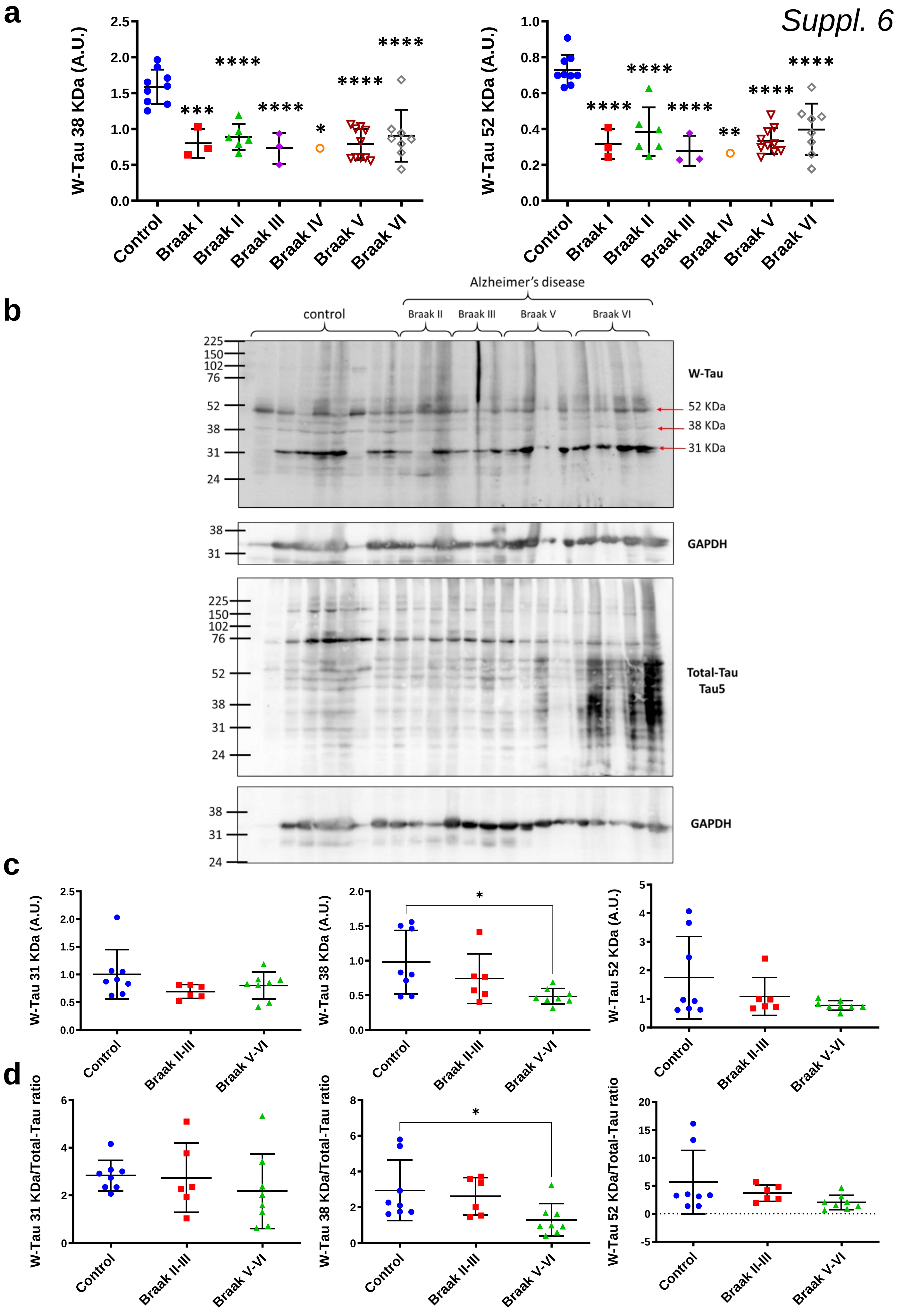

a
W-Tau 38 KDa (A.U.)
W-Tau 52 KDa (A.U.)
Control
Braak I
Braak II
Braak III
Braak IV
Braak V
Braak VI
***
****
*
**
b
Alzheimer's disease
control
Braak II
Braak III
Braak V
Braak VI
W-Tau
52 KDa
38 KDa
31 KDa
GAPDH
Total-Tau
Tau5
225
150
102
76
52
38
31
24
c
W-Tau 31 KDa (A.U.)
W-Tau 38 KDa (A.U.)
W-Tau 52 KDa (A.U.)
Braak II-III
Braak V-VI
d
W-Tau 31 KDa/Total-Tau ratio
W-Tau 38 KDa/Total-Tau ratio
W-Tau 52 KDa/Total-Tau ratio

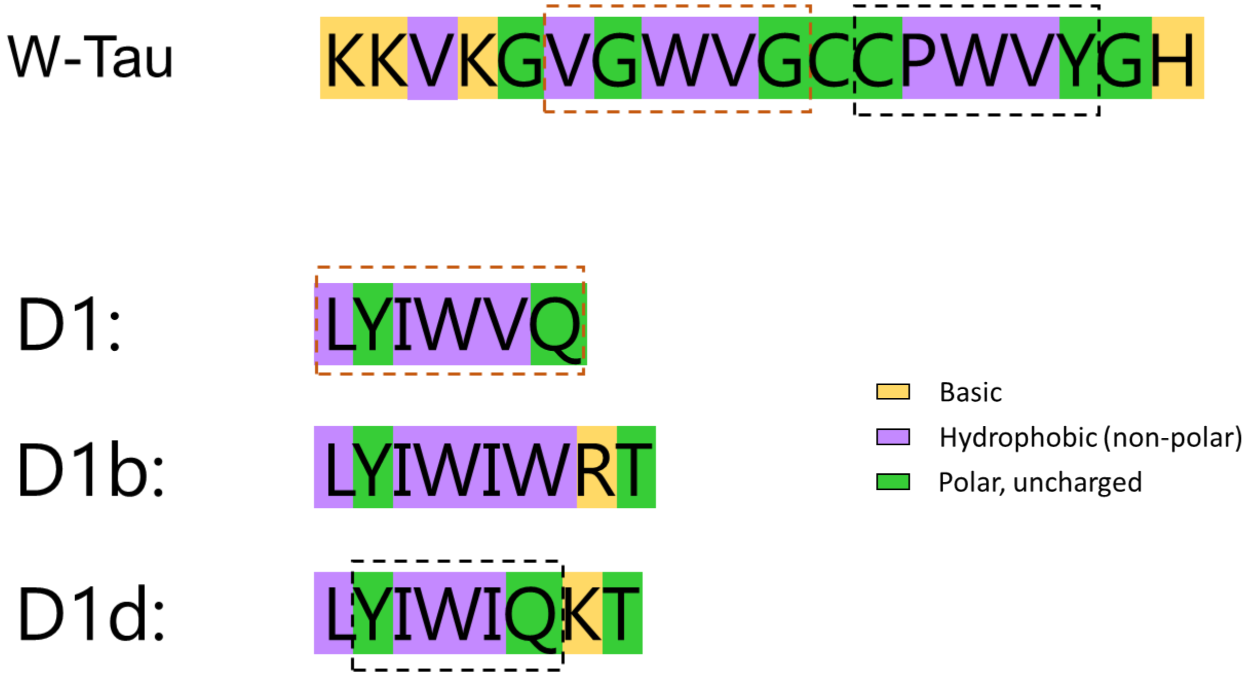
W-Tau
KKVKGVGWVGCCPWVYGH
D1:
LYIWVQ
D1b:
LYIWIWRT
D1d:
LYIWIQKT
Basic
Hydrophobic (non-polar)
Polar, uncharged

Supplementary Table 1. Information of brain samples from control non-demented subjects and AD patients classified according to their Braak stage.

Braak stage	Sex	Age	Hippocampal samples	Cortex samples
Control	M	41	qPCR, WB	WB
Control	F	58	qPCR, WB	qPCR, WB
Control	F	78	qPCR	WB
Control	M	43	qPCR, WB	WB
Control	M	56	qPCR, WB	WB
Control	F	49	qPCR, WB	WB
Control	M	84	qPCR	WB
Control	M	84		WB
Control	M	78	qPCR, WB	WB
Control	M	14	WB	
I	F	87		WB
I	M	77		WB
I	M	78		WB
II	M	87	qPCR	WB
II	M	80	qPCR	WB
II	F	85	qPCR	WB
II	M	84		WB
II	M	103		WB
II	F	82		WB
III	M	85	qPCR, WB	WB
III	M	85	qPCR, WB	WB
III	F	Not known	WB	
III	M	81		WB
IV	F	98		WB
V	F	87		WB
V	F	73	qPCR, WB	WB
V	F	88	qPCR, WB	WB
V	F	82	qPCR, WB	WB
V	M	87	qPCR, WB	WB
V	M	81	qPCR	WB
V	M	62	qPCR	WB
V	F	89	qPCR	WB
V	F	80		WB
V	F	84		WB
VI	M	68		WB
VI	M	76	qPCR, WB	WB
VI	M	88		WB
VI	F	86		WB
VI	F	81	qPCR, WB	WB
VI	M	92	qPCR	WB
VI	F	84	qPCR, WB	WB
VI	F	81	qPCR, WB	WB

Supplementary Table 2 Detailed information of the oligonucleotides used as primers for semi-quantitative, quantitative RT-PCR and cloning.

Name	Sequence
Oligos for semiquantitative RT-PCR	
TauA	ACCAGTTGACCTGAGCAAGG
TauB	GGTGGCCAGGTGGAAGTAAA
TauC	GACCAGCCTTTGTCCACTCA
TauD	AGCCAGTCAACACAGAAGCC
TauE	GACACCACTGGCGACTTGTA
TauG	CCATCATAAACCAGGAGGTGGC
TauH	GCGGCAGTGTGCAAATAGTC
GAPDH Fw	GAGAAGGCTGGGGCTCATTT
GAPDH Rv	AGTGATGGCATGGACTGTGG
Oligos for quantitative RT-PCR	
TIR-T-fw	CATAAACCAGGAGGTGGCCAG
TIR-T-rv	CACCCTACCCCCTTTACCTTTT
MAPT-E11-E13-fw	GTCGAAGATTGGGTCCCTGG
MAPT-E11-E13-rv	GACACCACTGGCGACTTGTA
Oligos for cloning	
ET-T-PacI	GTTTAATTAATCAATTTCCTCCGCCAGGGACGTGGG
TIR-T-PacI	AATTAATTAATGCCCATATACCCAAGGGCAGC
A22	CAAGATCTCAATTTCCTCCGCCAGGGACGTGGG
Tau-Nt	ATGGCTGAGCCCCGCCAGGAG
TIR-T-BglII	GAGATCTTAATGCCCATATACCCAAGGGCAGC
ET-T-PacI	GTTTAATTAATCAATTTCCTCCGCCAGGGACGTGGG
TIR-T-PacI	AATTAATTAATGCCCATATACCCAAGGGCAGC
Tau-Nt	ATGGCTGAGCCCCGCCAGGAG
TIR-T-BglII	GAGATCTTAATGCCCATATACCCAAGGGCAGC
TAU-PRK172 fw	GCGGATCCATATGGCTGAGCCC
TIR-T-pRKpWPI rv	GCGAATTCTTAATGCCCATATACCCAAGGG
ET-T-pRKpWPI rv	GTTCTGAATTCTTAATTAATCAATTTCCTCCGCCAGG
Extra oligos for sequencing	
A22	CAAGATCTCAATTTCCTCCGCCAGGGACGTGGG
A1	GGCGAATTCGGATCCTATGGCTGAGCCCCGCCAGGAG
A4	GCTGCTCCCCGCGGTGTG
Tau-Ct	ACCCTGCTTGGCCAGGGAGGC
Tau R1 Rv	CCGCTGTTGGAGTGCTCTTA
Tau 447 Rv	CGTTTTACCATCAGCCCCCT
pSGTau-fw	CTCACTATAGGGCGAATTCATG
pSGTau-rv	AGCGGAAGAGTCTAGAGTCG

Supplementary Table 3. Number of healthy brain samples and donors per brain region within GTEx that were RNA-seq analyzed. Venn diagram shows number of individuals for each sample region.

Brain Region	Number of samples	Duplicated samples	Triplicated samples	Number of donors
Frontal cortex	134	3	2	127
Dorsolateral prefrontal cortex	120	1	4	115
Hippocampus	109	3	1	100

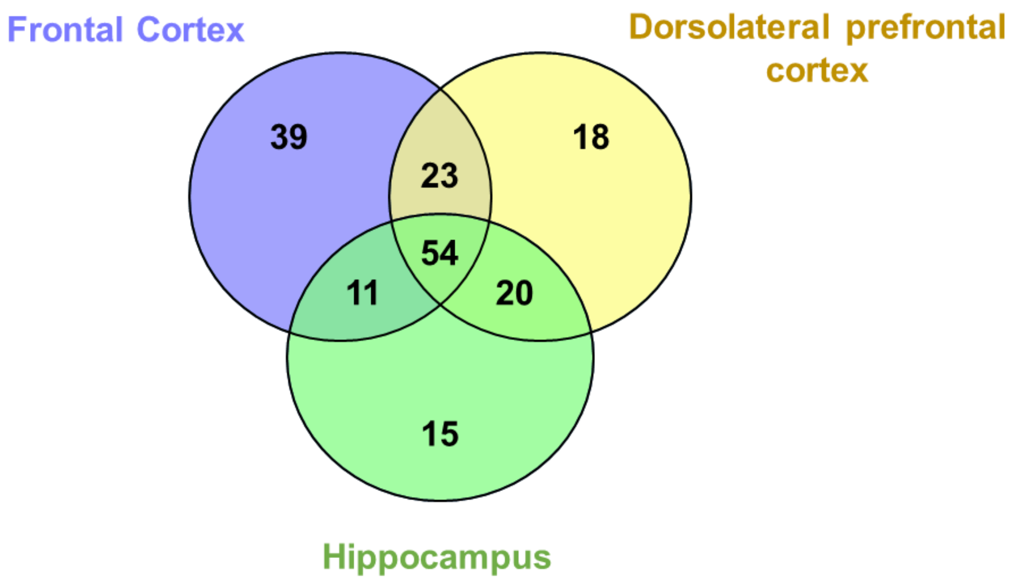

Supplementary Table 4. Amino acid composition of different Tau isoforms and calculated extinction coefficient (ε) for different Tau isoforms, according to their amino acid composition. Differences on tryptophan (W) and tyrosine (Y) are highlighted in green.

Amino acid	T42	T30	TIR42	TIR30	ET42	ET30
Alanine (A)	34	27	27	20	27	20
Arginine (R)	14	14	12	12	12	12
Asparagine (N)	11	8	9	6	9	6
Aspartate (D)	29	23	24	18	24	18
Cysteine (C)	2	1	4	3	2	1
Glutamine (Q)	19	14	17	12	17	12
Glutamate (E)	27	18	23	14	23	14
Glycine (G)	49	40	49	40	45	36
Histidine (H)	12	10	10	8	9	7
Isoleucine (I)	15	11	12	8	12	8
Leucine (L)	21	17	15	11	15	11
Lysine (K)	44	37	40	33	37	30
Methionine (M)	6	6	5	5	5	5
Phenilalanine (F)	3	3	2	2	2	2
Proline (P)	43	34	41	32	40	31
Serine (S)	45	36	35	26	35	26
Threonine (T)	35	26	29	20	29	20
Tryptophan (W)	0	0	2	2	0	0
Tyrosine (Y)	5	5	5	5	4	4
Valine (V)	27	22	25	20	21	16
**Total**	**441**	**352**	**386**	**297**	**368**	**279**

Tau isoform	Extinction Coefficient (ε) ($M^{-1} \cdot cm^{-1}$)
T42	7450
T30	7450
W-T42	18450
W-T30	18450
ET-T42	5960
ET-T30	5960

Supplementary Table 5. Detailed information of the oligonucleotides used for each semi-quantitative RT-PCR and the length of the corresponding amplicons.

PCR number	Forward primer	Reverse primer	Amplicon length
1	TauB	TauC	188
2	TauB	TauD	232
3	TauG	TauC	204
4	TauG	TauD	248
5	TauA	TauC	255
6	TauA	TauD	299
7	TauH	TauC	280
8	TauH	TauD	324
9	TauA	TauE	256
10	TauB	TauE	200
11	TauG	TauE	216
12	TauH	TauE	281
13	GAPDH Fw	GAPDH Rv	231
